# The Global Burden of Vascular Intestinal Disorders in 204 Countries and Territories From 1990 to 2019: Global Burden of Diseases Study

**DOI:** 10.3389/ijph.2023.1606297

**Published:** 2023-09-26

**Authors:** Tianxiang Jiang, Zhaolun Cai, Mingchun Mu, Zhou Zhao, Chaoyong Shen, Bo Zhang

**Affiliations:** ^1^ Department of General Surgery, West China Hospital, Sichuan University, Chengdu, China; ^2^ Gastric Cancer Center, West China Hospital, Sichuan University, Chengdu, China; ^3^ Department of Gastrointestinal Cancer Center, Chongqing University Cancer Hospital, Chongqing, China

**Keywords:** global burden of diseases, mesenteric ischemia, ischemic colitis, angiodysplasia, intestines

## Abstract

**Objectives:** Assess the prevalence, mortality, and disability-adjusted life years (DALYs) of vascular intestinal disorders (VID) from 1990 to 2019.

**Methods:** This study conducted a secondary data analysis utilizing the Global Burden of Diseases Study 2019. The prevalence, mortality and DALYs of VID were analyzed by sex, age and socio-demographic index (SDI), respectively. Analyses were performed by using R software.

**Results:** Globally, the number of prevalent VID cases increased from 100,158 (95% uncertainty interval: 89,428–114,013) in 1,990–175,740 (157,941–198,969) in 2019. However, the age-standardized rates (ASR) of VID prevalence declined from 2.47 (95% uncertainty interval: 2.24–2.76) per 100,000 population to 2.21 (1.98–2.48) per 100,000 population between 1990 and 2019. Furthermore, the ASR of mortality also decreased from 1990 to 2019. Between 1990 and 2019, the regions with high and high-middle level exhibited the highest diseases burden.

**Conclusion:** Globally, the diseases burden associated with VID demonstrated a decline from 1990 to 2019. However, concerted efforts are still required to enhance measures to combat VID within countries categorized as high and high-middle SDI.

## Introduction

Vascular intestinal disorders (VID) encompass conditions like mesenteric ischemia [1], ischemic colitis [2], and angiodysplasia of the intestine [3], which present a wide range of clinical symptoms. These manifestations are non-specific and include abdominal pain, diarrhea, bleeding, nausea, vomiting, and weight loss [4]. In severe cases of intestinal ischemia, patients may exhibit signs of peritonitis, such as abdominal distension, guarding, and hypotension, which can be life-threatening [5]. Regrettably, due to the vague nature of these symptoms, diagnosing VID is often delayed, leading to a significant increase in morbidity and mortality rates, which can range from 60% to 80% [6]. Moreover, VID negatively impacts the quality of life for affected patients [7, 8], affecting their ability to work and putting additional strain on their families and society as a whole [9, 10].

Mesenteric ischemia, a condition characterized by inadequate blood supply to the intestines, arises from various causes. About half of cases are due to arterial embolism originating from the heart, while the rest are attributed to arterial thrombosis (associated with atherosclerosis), venous thrombosis (linked to coagulation disorders), and non-occlusive ischemia (caused by factors like hypovolemia, hypotension, and reduced cardiac output) [1, 11]. Furthermore, ischemic colitis has been linked to specific drugs, such as cocaine [12] and tegaserod [13], as well as colonoscopy procedures [14]. Studies have also reported associations between ischemic colitis and coronary artery disease, as well as end-stage renal disease requiring dialysis [15–17]. The development of intestinal angiodysplasia has been associated with vascular endothelial growth factor [18] and von Willebrand facto [19, 20]. To address VID, treatment approaches encompass various strategies. These include conservative measures such as heparin, aspirin, oxygen therapy, proton pump inhibitors, and drugs targeting atherosclerosis, hypovolemia, hypotension, and decreased cardiac output. Catheter-based endovascular revascularization techniques, such as angioplasty, stenting, thromboaspiration, intra-arterial vasodilation, and thrombolysis, are employed to manage arterial stenosis or emboli in the digestive system. Surgical interventions, such as exploratory laparotomy, surgical revascularization, and necrotizing bowel resection, are also considered [21, 22].

A study in America reported that the adjusted prevalence of ischemic colitis increased significantly from 6.1 cases per 100,000 person-years in 1976–1980 to 22.9 cases per 100,000 person-years in 2005–2009(16). In Sweden, a population-based estimate for acute mesenteric ischemia diagnosed through autopsy or surgery in the Malmö population between 1970 and 1982 showed a prevalence of 12.9 cases per 100,000 person-years [23]. Similarly, a study in the Kuopio population in Finland from 2009 to 2013 revealed a prevalence of 7.3 cases per 100,000 person-years for acute mesenteric ischemia, with individuals aged 75 years or older having a higher prevalence of 48.3 cases per 100,000 person-years [24]. Linear regression analysis of mortality for acute mesenteric infarction from 1954 to 2012 showed a significant decrease in in-hospital mortality during this period (*p* = 0.027). However, from 2002 to 2012, the decline in mortality was not statistically significant (*p* = 0.78) [25]. A meta-analysis examined studies from high-income countries between 1970 and 2013, which reported an average annual diagnosis rate of 6.2 new cases of acute mesenteric ischemia per 100,000 inhabitants (confidence interval 1.9–12.9) [26]. Despite these findings, the geographical coverage of these studies remains limited, and there is a lack of reported data on the global burden of VID. In addition, the burden of VID has not been analyzed in relation to the socio-demographic index (SDI) of each country. SDI is a composite indicator of a country’s lagged distribution of *per capita* income, average years of schooling, and the fertility rate of women under 25 years of age [27, 28]. Therefore, we aim to assess the burden of VID over time, considering different geographical locations, sexes, age groups, and SDI levels. To achieve this, we utilized information provided in the Global Burden of Disease Study (GBD) 2019(28). By conducting this analysis, we seek to provide crucial insights into the burden of VID and ultimately contribute to evidence-based strategies for effective disease prevention and management.

## Methods

### Overview

The GBD 2019 database is a groundbreaking analysis that includes a wide range of diseases and injuries, causes of death and risk factors across the world [28]. It comprises data from 1990 to 2019, encompassing 21 regions and 204 countries and territories. The main objectives, methodology, and structure of GBD 2019 have been thoroughly documented in previous reports [28, 29].

The GBD 2019 study adopted a comprehensive approach to assess the non-fatal burden of VID. It utilized a wide range of data sources, including hospital discharge records, claims data, peer-reviewed publications, and household surveys. These carefully collected data served as the foundation for estimating the prevalence of VID in different geographical locations, years, age groups, and sexes with utilizing of the disease model-Bayesian meta-regression 2.1 (DisMod-MR 2.1). To measure the level of seriousness of VID in a quantitative manner, specific disability weights were assigned to each individual condition. By multiplying the prevalence of each sequela with its corresponding disability weight, the study calculated the years lived with disability (YLD), providing a robust estimate of the burden posed by VID.

In order to determine the fatal burden of VID, GBD 2019 undertook an extensive data collection process, which involved gathering data from vital registration and vital registration samples from databases of reasons for death. The study utilized standard cause of death integration modeling methods to accurately estimate the mortality attributable to VID, considering factors such as location, year, age, and sex [28]. To calculate Years of Life Lost (YLL) due to VID, the study used standardized life expectancy worldwide and the death population based on age. The assessment of DALYs served as a crucial measure of the overall burden imposed by VID. The DALYs were determined by combining YLD and YLL. To address uncertainties in the data, a posterior distribution with values of 2.5% and 97.5% is defined as uncertainty interval.

### Disease Definition

VID is accurately characterized by GBD 2019 as a vascular abnormality that occurs in the intestines and affects both the small intestine and/or the colon. This comprehensive definition covered various conditions, such as ischemic disorders and vascular malformations. The International Classification of Diseases (ICD-10) associated with VID is shown in Supplementary Table S1, which provides detailed information.

### Research Dimension

GBD defined SDI to assess socioeconomic conditions that influence health outcomes across geographic locations [27, 28]. Measured on a scale ranging from 0 (worst) to 100 (best), the SDI considers key factors such as national-level income, average years of schooling for individuals aged 15 and above, and the total fertility rate for females under the age of 25(27). In GBD 2019, a total of 204 countries and territories were included, further grouped into 21 regions for comprehensive analysis and comparison [28]. Accordingly, the SDI categorizes these countries and regions into five classes from low to high. Supplementary Table S2 provides a comprehensive list of specific sites covered by each region.

### Source of Data and Statistical Analysis

This study involves a re-analysis of publicly available data on the disease burden related to VID on a global scale. The Input Data and Methodological Summary for VID by GBD 2019 Diseases and Injuries Collaborators (licensed under CC BY 4.0) are provided in Supplementary Materials S1 [28]. The data extracted from GBD 2019 includes the numbers, age-standardized rate (ASR), and percentage change in ASR between 1990 and 2019. Prevalence, mortality, and DALYs rates are expressed as the cases, deaths, and DALYs per 100,000 population. To examine trends in ASR over the specified time period, the study employed the estimated annual percentage change (EAPC) method proposed by Hankey et al. [30]. This involved fitting a regression line to the natural logarithm of the ratio, with *y = ln(ASR)* and *x = the calendar year*. The EAPC was then calculated as *100 ×* (*exp(β) - 1*), and its 95% uncertainty interval (CI) was derived from the linear regression model. Additionally, the study assessed the associations between the SDI and the burden of VID using Pearson correlation tests. The R software (version 4.0.3, R Core Team) was used for statistical analyses.

## Results

### Prevalence

**FIGURE 1:**
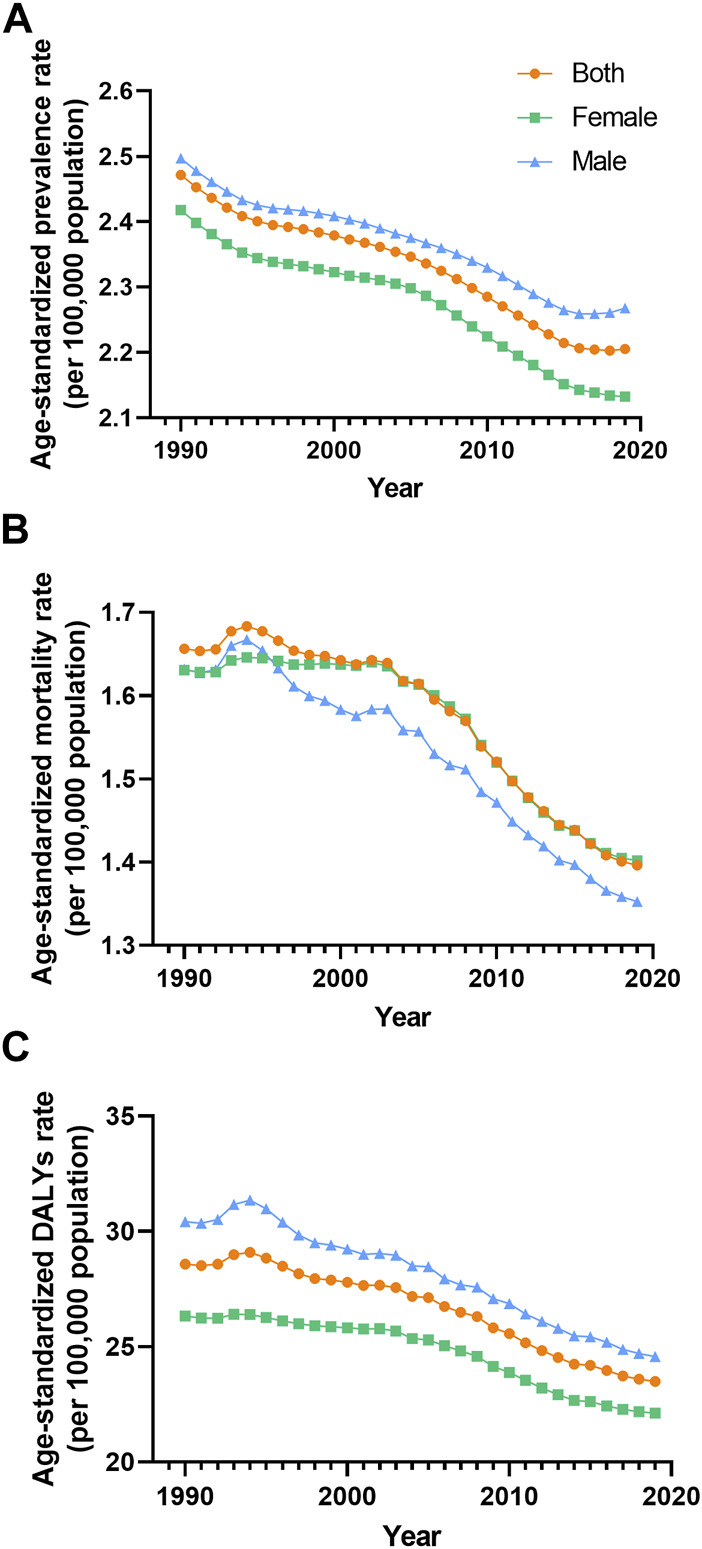
Prevalence **(A)**, mortality **(B)** and disability-adjusted life years **(C)** agestandardized rate of vascular intestinal disorders by sex Global 1990–2019. ASR, age-standardized rate; VID, vascular intestinal disorders.

**FIGURE 2:**
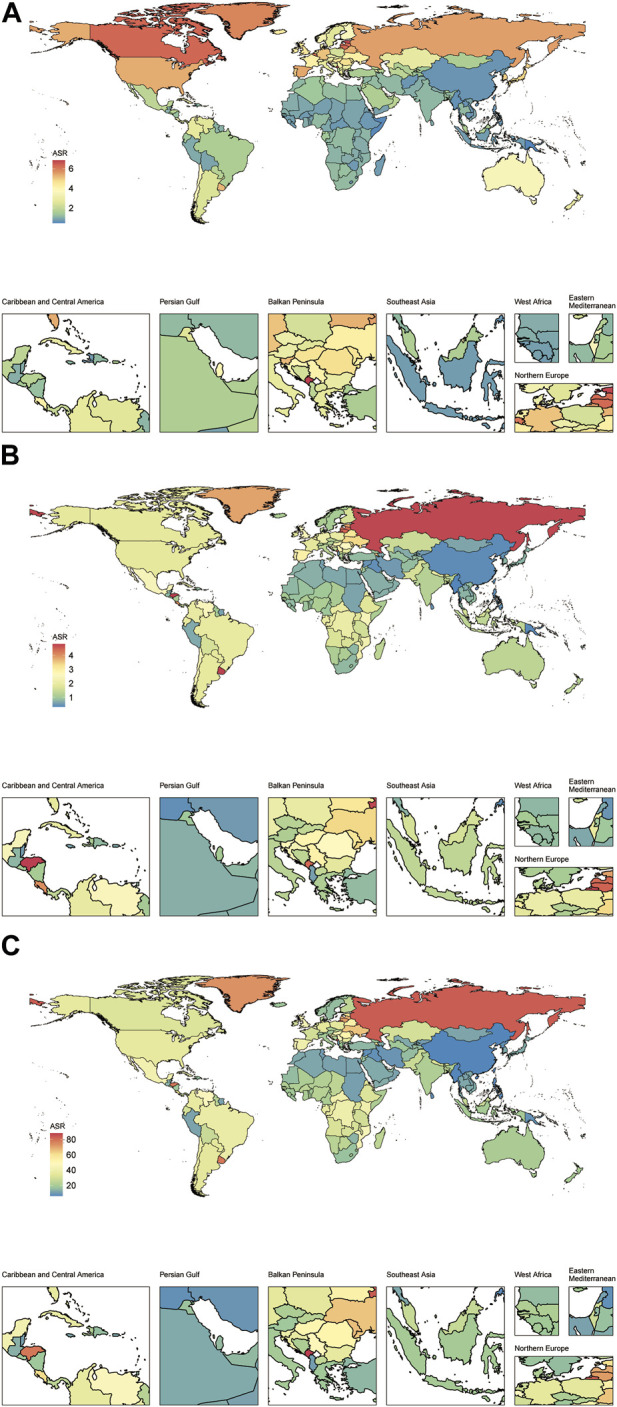
The prevalence **(A)**, mortality **(B)** and disability-adjusted life years **(C)** agestandardized rate of vascular intestinal disorders for both sexes in 204 countries and territories. Global, 2019. ASR, age-standardized rate; VID, vascular intestinal disorders.

**FIGURE 3:**
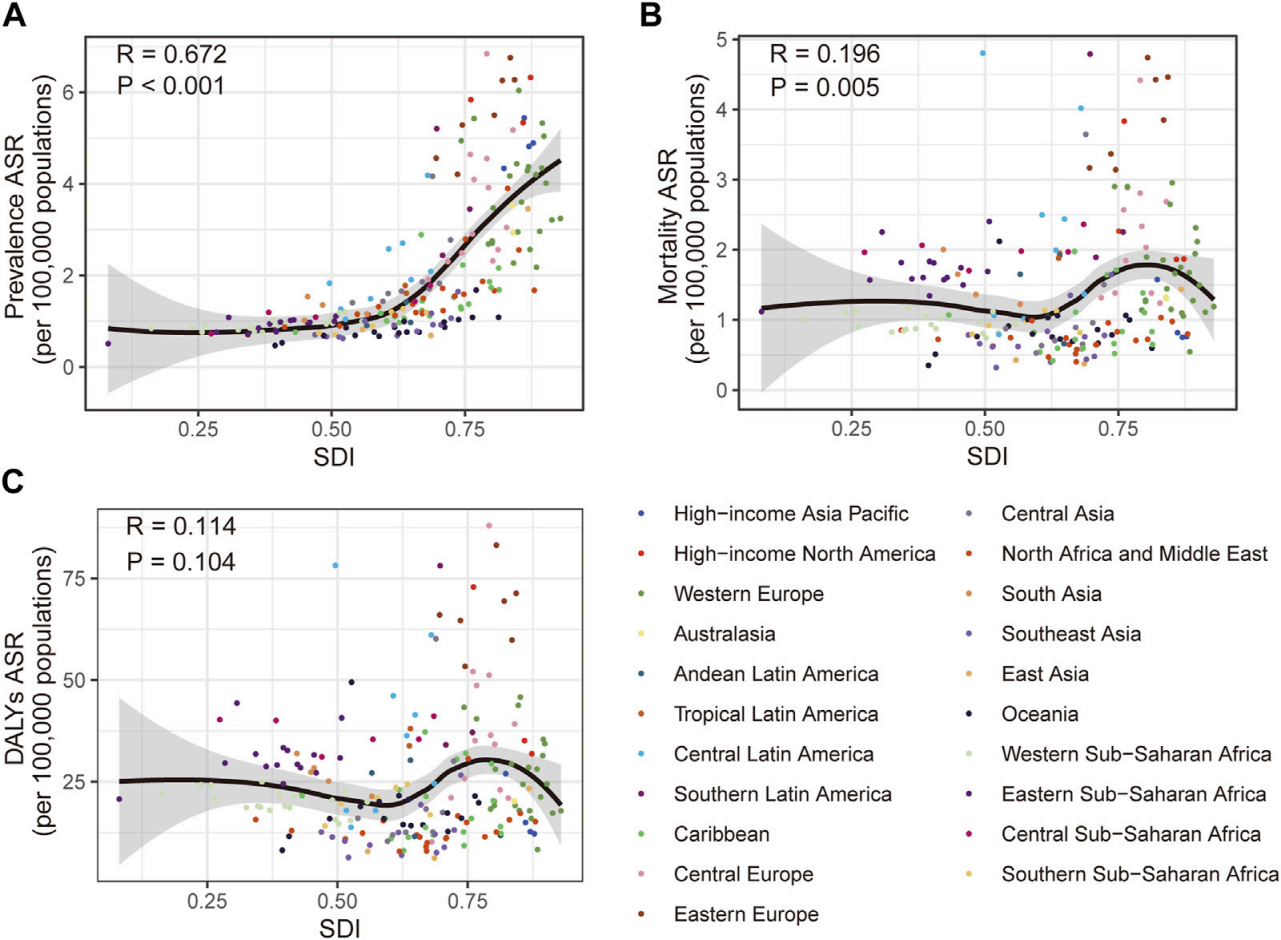
Correlation of the prevalence **(A)**, mortality **(B)** and disability-adjusted life years **(C)** age-standardized rate of vascular intestinal disorders with different socio-demographic index in 204 countries and territories. Global, 2019. DALYs, disability-adjusted life years; ASR, age-standardized rate; SDI, socio-demographic index.

In 2019, the estimated global prevalence of VID was 175,740 cases (95% uncertainty interval 157,941–198,969) with an ASR of 2.21 (1.98–2.48) per 100,000 population. From 1990 to 2019, the EAPC of the ASR showed a decline of −0.39% (−0.42 to −0.37) (Table 1). The analysis of trends indicated a decrease in the global prevalence ASR of VID from 1990 to 2018, followed by a slight increase from 2018 to 2019 (Figure 1A). Among the 21 regions, High-income North America [ASR 5.44 (4.98–5.98)] and High-income Asia Pacific [ASR 4.83 (4.07–5.84)] had the highest prevalence ASR of VID per 100,000 population in 2019. In contrast, Oceania [ASR 0.57 (0.46–0.72)] and East Asia [ASR 0.75 (0.64–0.89)] (Table 1; Supplementary Figure S1A) reported the lowest prevalence in 2019. Notably, Andean Latin America showed the highest increase in prevalence ASR from 1990 to 2019 [EAPC 1.73% (1.69–1.76)] (Table 1; Supplementary Figure S1B), while Tropical Latin America demonstrated the largest decrease [EAPC -1.38% (−1.57 to −1.18)]. In 2019, country-level prevalence ASR ranged between 0.46 and 6.84 per 100,000 population. Montenegro and Estonia exhibited the highest prevalence ASR (Figures 2A, 3A; Supplementary Table S3). Moreover, changes in prevalence ASR over the 30-year period varied across the country-level, with Equatorial Guinea reporting the largest increase and Sweden experiencing the largest decrease (Supplementary Figure S2A; Supplementary Table S3).

**TABLE 1:** Prevalence, mortality and disability-adjusted life years for vascular intestinal disorders in 2019 and estimated annual percentage change in the age-standardized rate from Global, 1990 to 2019.

Measure	Prevalence (95% UI)			Mortality (95% UI)			DALYs (95% UI)		
	Cases (95% UI)	ASR (95% UI)	EAPC (95% UI)	Deaths (95% UI)	ASR (95% UI)	EAPC (95% UI)	Years (95% UI)	ASR (95% UI)	EAPC (95% UI)
Global	175,740 (157,941–198,969)	2.21 (1.98–2.48)	−0.39% (−0.42 to −0.37)	106,576 (95,113–116,428)	1.4 (1.24–1.53)	−0.68% (−0.77 to −0.59)	1,872,339 (1,722,038–2,034,447)	23.49 (21.58–25.59)	−0.78% (−0.85 to −0.71)
High SDI	77,381 (70,783–84,766)	4.57 (4.1–5.16)	−0.31% (−0.32 to −0.29)	34,333 (29,863–37,570)	1.59 (1.41–1.73)	−1.14% (−1.27 to −1.02)	519,308 (474,724–551,923)	27.42 (25.36–28.96)	−1.07% (−1.16 to −0.97)
High-middle SDI	46,965 (42,995–51,790)	2.47 (2.25–2.74)	−0.18% (−0.25 to −0.12)	35,058 (31,532–37,745)	1.78 (1.6–1.92)	−0.26% (−0.4 to −0.12)	583,263 (537,823–621,728)	29.17 (26.87–31.07)	−0.66% (−0.8 to −0.52)
Middle SDI	26,405 (22,564–31,877)	1.1 (0.95–1.31)	0.5% (0.45–0.56)	17,714 (15,926–19,427)	0.87 (0.78–0.96)	−0.2% (−0.24 to −0.16)	334,507 (307,149–363,488)	14.49 (13.24–15.78)	−0.41% (−0.43 to −0.38)
Low-middle SDI	16,962 (14,197–21,200)	1.08 (0.92–1.32)	1.13% (0.99–1.26)	13,662 (11,125–17,070)	1.19 (0.98–1.47)	0.23% (0.17–0.28)	287,609 (232,710–360,988)	21.62 (17.58–27.02)	0.13% (0.09–0.16)
Low SDI	7,944 (6,185–10,497)	0.91 (0.77–1.12)	0.82% (0.68–0.95)	5,756 (4,717–6,896)	1.37 (1.13–1.61)	0.02% (−0.02–0.06)	146,732 (116,415–185,308)	25.73 (21.09–31.05)	−0.22% (−0.26 to −0.17)
Andean Latin America	620 (536–741)	1.08 (0.94–1.27)	1.73% (1.69–1.76)	587 (479–715)	1.1 (0.89–1.34)	−0.23% (−0.38 to −0.07)	10,477 (8,623–12,696)	18.79 (15.49–22.84)	−1.05% (−1.28 to −0.82)
Australasia	1,639 (1,483–1827)	3.43 (3.08–3.88)	−0.18% (−0.24 to −0.13)	740 (615–842)	1.32 (1.11–1.5)	−1.25% (−1.31 to −1.19)	10,346 (8,968–11,527)	20.28 (17.83–22.53)	−1.54% (−1.61 to −1.47)
Caribbean	923 (818–1,058)	1.83 (1.62–2.1)	0.83% (0.8–0.86)	660 (544–800)	1.28 (1.05–1.55)	−0.63% (−0.82 to −0.45)	12,529 (10,427–15,182)	24.36 (20.24–29.59)	−0.71% (−0.9 to −0.53)
Central Asia	1722 (1,435–2,139)	2.11 (1.81–2.55)	1.36% (1.19–1.53)	650 (584–719)	1.17 (1.04–1.31)	0.65% (0.51–0.8)	14,238 (12,816–15,824)	20.99 (18.9–23.17)	0.16% (0.02–0.3)
Central Europe	6,004 (5,508–6,542)	3.31 (2.98–3.73)	0.73% (0.57–0.88)	4,791 (4,205–5,366)	2.13 (1.87–2.39)	0.59% (0.23–0.95)	80,015 (70,998–89,933)	37.67 (33.46–42.28)	0.14% (−0.15–0.44)
Central Latin America	5,036 (4,516–5,743)	2.15 (1.94–2.43)	0.13% (0.09–0.18)	5,034 (4,284–5,826)	2.26 (1.92–2.62)	−0.21% (−0.27 to −0.15)	91,301 (78,161–104,954)	39.36 (33.67–45.24)	−0.4% (−0.44 to −0.35)
Central Sub-Saharan Africa	1,001 (800–1,276)	1.16 (1.01–1.36)	0.56% (0.38–0.74)	814 (501–1,243)	1.99 (1.24–2.93)	−0.91% (−1.08 to −0.75)	21,400 (13,134–33,494)	37.93 (23.36–58.06)	−1.02% (−1.19 to −0.84)
East Asia	14,389 (12,026–17,453)	0.75 (0.64–0.89)	0.16% (0.06–0.26)	7,113 (5,860–8,126)	0.4 (0.33–0.46)	−0.34% (−0.44 to −0.25)	130,067 (106,734–149,275)	6.64 (5.48–7.57)	−0.67% (−0.75 to −0.59)
Eastern Europe	16,740 (15,646–17,992)	5.23 (4.81–5.72)	0.72% (0.63–0.82)	15,192 (13,584–16,817)	4.32 (3.87–4.78)	1.43% (1.24–1.62)	263,459 (236,239–291,640)	76.94 (68.97–85.29)	0.95% (0.75–1.16)
Eastern Sub-Saharan Africa	2,593 (2002–3,408)	0.9 (0.76–1.09)	1.16% (1.04–1.29)	2,278 (1,639–2,826)	1.8 (1.29–2.25)	0.46% (0.38–0.54)	54,424 (40,217–66,452)	32.29 (23.33–40.05)	0.28% (0.19–0.36)
High-income Asia Pacific	15,628 (13,565–18,080)	4.83 (4.07–5.84)	0.42% (0.31–0.53)	4,402 (3,464–5,067)	0.75 (0.61–0.84)	0.46% (0.29–0.62)	58,402 (50,095–63,696)	12.59 (11.2–13.7)	0% (−0.13 to 0.13)
High-income North America	30,981 (28,644–33,647)	5.44 (4.98–5.98)	−0.75% (−0.77 to −0.73)	12,498 (11,113–13,432)	1.86 (1.68–1.99)	−1.06% (−1.27 to −0.84)	211,893 (196,390–223,239)	34.78 (32.41–36.53)	−1.01% (−1.17 to −0.84)
North Africa and Middle East	6,532 (5,412–8,058)	1.34 (1.14–1.6)	1.69% (1.64–1.75)	2,365 (2039–2,708)	0.68 (0.59–0.78)	−0.31% (−0.39 to −0.24)	48,945 (42,093–56,445)	11.95 (10.33–13.67)	−0.53% (−0.57 to −0.49)
Oceania	60 (47–79)	0.57 (0.46–0.72)	0.55% (0.49–0.62)	28 (22–36)	0.49 (0.4–0.6)	0.26% (0.23–0.29)	842 (641–1,096)	10.59 (8.36–13.32)	0.39% (0.33–0.45)
South Asia	18,873 (15,509–24,221)	1.15 (0.97–1.44)	1.15% (1.01–1.29)	14,580 (10,744–19,539)	1.28 (0.96–1.68)	−0.1% (−0.24 to 0.05)	298,900 (217,378–406,713)	22.33 (16.43–30.1)	−0.15% (−0.25 to −0.05)
Southeast Asia	5,421 (4,518–6,654)	0.89 (0.77–1.07)	1.07% (1.02–1.13)	4,134 (3,571–4,706)	0.9 (0.77–1.02)	−0.01% (−0.07 to 0.05)	73,414 (64,359–83,289)	13.76 (12.06–15.67)	−0.24% (−0.3 to −0.18)
Southern Latin America	2,391 (2,178–2,641)	2.91 (2.63–3.24)	0.92% (0.8–1.03)	1889 (1,678–2097)	2.2 (1.96–2.45)	−0.87% (−1 to −0.73)	31,543 (28,747–34,701)	37.85 (34.51–41.62)	−0.95% (−1.05 to −0.85)
Southern Sub-Saharan Africa	786 (637–1,014)	1.1 (0.91–1.38)	0.35% (0.26–0 .45)	409 (359–455)	0.83 (0.74–0.93)	0.51% (0.35–0.68)	9,973 (8,797–11,181)	16.89 (14.88–18.74)	0.3% (0.11–0.48)
Tropical Latin America	3,860 (3,427–4,432)	1.62 (1.44–1.85)	−1.38% (−1.57 to −1.18)	4,497 (4,069–4,976)	1.95 (1.75–2.16)	−1.5% (−1.56 to −1.43)	91,117 (84,334–98,288)	37.94 (35.04–41)	−1.6% (−1.68 to −1.52)
Western Europe	36,500 (33,650–39,678)	4.2 (3.81–4.66)	0.04% (−0.08–0.15)	22,141 (19,269–24,540)	2.01 (1.78–2.21)	−0.91% (−1.08 to −0.75)	299,807 (271,457–323,892)	31.42 (28.79–33.67)	−0.99% (−1.12 to −0.86)
Western Sub-Saharan Africa	4,046 (3,042–5,427)	1.04 (0.86–1.3)	0.52% (0.48–0.57)	1774 (1,353–2,298)	1.03 (0.8–1.37)	0.74% (0.64–0.84)	59,247 (44,506–79,067)	21.72 (16.57–28.23)	0.06% (0–0.12)

UI, uncertainty interval; DALYs, disability-adjusted life years; ASR, age-standardized rates; EAPC, estimated annual percentage changes.

### Mortality

In 2019, VID were responsible for an estimated 106,576 global deaths (95,113–116,428) with an ASR of 1.4 (1.24–1.53) per 100,000 population. The EAPC of the ASR from 1990 to 2019 was −0.68% (−0.77 to −0.59) (Table 1). Analyzing the trends, the global mortality ASR of VID increased from 1991 to 1994, followed by a slight decline from 1994 to 2001. Subsequently, it slightly increased from 2001 to 2002 before gradually declining from 2002 to 2019 (Figure 1B). Among the 21 regions, Eastern Europe [ASR 4.32 (3.87–4.78)] reported the highest mortality ASR of VID in 2019 per 100,000 population, while East Asia [ASR 0.4 (0.33–0.46)] and Oceania [ASR 0.49 (0.4–0.6)] had the lowest mortality ASR (Table 1; Supplementary Figure S1C). Notably, Eastern Europe exhibited the largest increase in mortality ASR from 1990 to 2019 [EAPC 1.43% (1.24–1.62)] (Table 1; Supplementary Figure S1D), while the most significant decrease was observed in Tropical Latin America [EAPC −1.5% (−1.56 to −1.43)]. At the country-level, the mortality ASR of VID in 2019 ranged from 0.32 to 4.80 per 100,000 population. At the country level, the highest mortality ASR are found in Honduras, Uruguay, and the Russian Federation (Figures 2B, 3B; Supplementary Table S3). Moreover, changes in mortality ASR over the 30 year period varied across the country-level, with the largest increase in Taiwan (Province of China) and the most significant decrease in Bahrain (Supplementary Figure S2B; Supplementary Table S4).

### DALYs

In 2019, the global burden of VID measured in DALYs was estimated to be 1,872,339 (1,722,038 to 2,034,447) with an ASR of 23.49 (21.58–25.59) per 100,000 population. The EAPC of the DALYs ASR from 1990 to 2019 was −0.78% (−0.85 to −0.71) (Table 1). Analyzing the trends (Figure 1C), the global DALYs ASR of VID increased from 1991 to 1994, followed by a gradual decrease from 1994 to 2019. 2019 VID DALYs ASR Maximum and Minimum regions (Table 1), as well as those with the most significant decrease in DALYs ASR over the 30 years period (Supplementary Figures S1E, F), were consistent with VID mortality. In 2019, country-level DALYs ASR ranged between 6.24 and 87.99 per 100,000 population. Montenegro and the Russian Federation showed the maximum value of DALYs ASR (Figure 2C; Supplementary Table S5). Furthermore, over the 30-year period, Bahrain experienced the maximum reduction in the DALYs ASR, while Taiwan (Province of China) showed the largest increase (Supplementary Figure S2C; Supplementary Table S5).

### Age- and Sex-Related Burden

**FIGURE 4:**
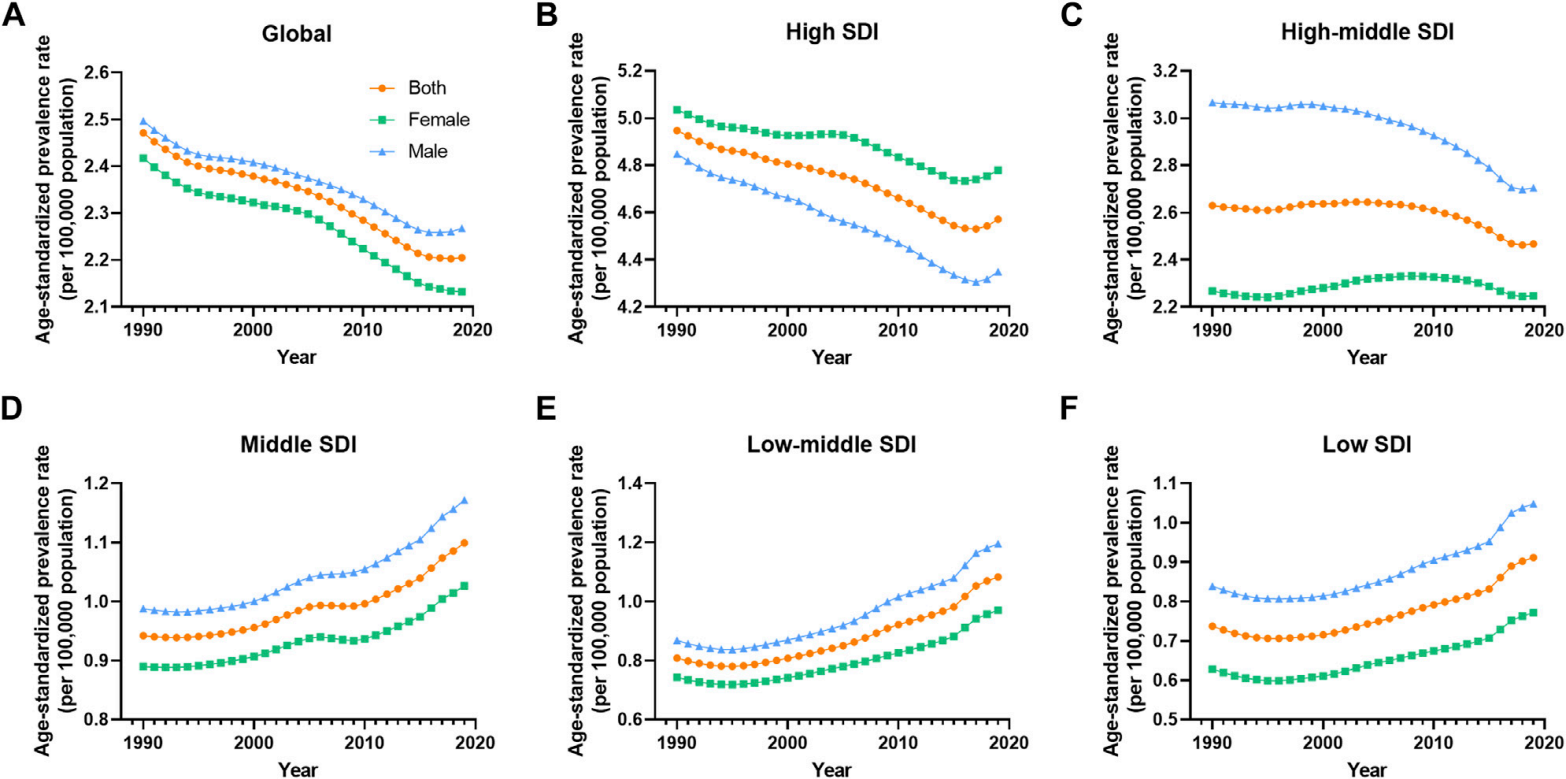
Prevalence age-standardized rate of vascular intestinal disorders by sex and socio-demographic index quintile. Global, 1990–2019. **(A)** Global, **(B)** High SDI, **(C)** High-middle SDI, **(D)** Middle SDI, **(E)** Low-middle SDI, **(F)** Low SDI. ASR, age-standardized rate; VID, vascular intestinal disorders, SDI, socio-demographic index.

Between 1990 and 2019, the global prevalence and DALYs ASR of VID consistently showed higher values in males compared to females. However, the mortality ASR for males was lower than that for females in most years, except for the period between 1990 and 1995 (Figures 1A–C). Across most SDI quintiles, males exhibited higher prevalence ASR of VID than females from 1990 to 2019, with the exception of the high SDI quintile (Figure 4). In 2019, except for High-income North America, High-income Asia Pacific, and Southeast Asia, all regions displayed higher prevalence ASR among males compared to females (Supplementary Figure S1A). Eastern Europe showed the highest increases in mortality and DALYs ASR for females, while Andean Latin America reported the highest increase in prevalence ASR (Supplementary Figures S1B, D, F). Across all age groups, the burden of VID raised with age. It is worth noting that the increase in mortality is particularly sharp at ages 85 and above (Supplementary Figure S3). Additionally, in the younger age groups, the global burden was higher in males, while at ages 75 and above, these rates were higher in females (Supplementary Figure S3).

### SDI-Related Burden

**FIGURE 5:**
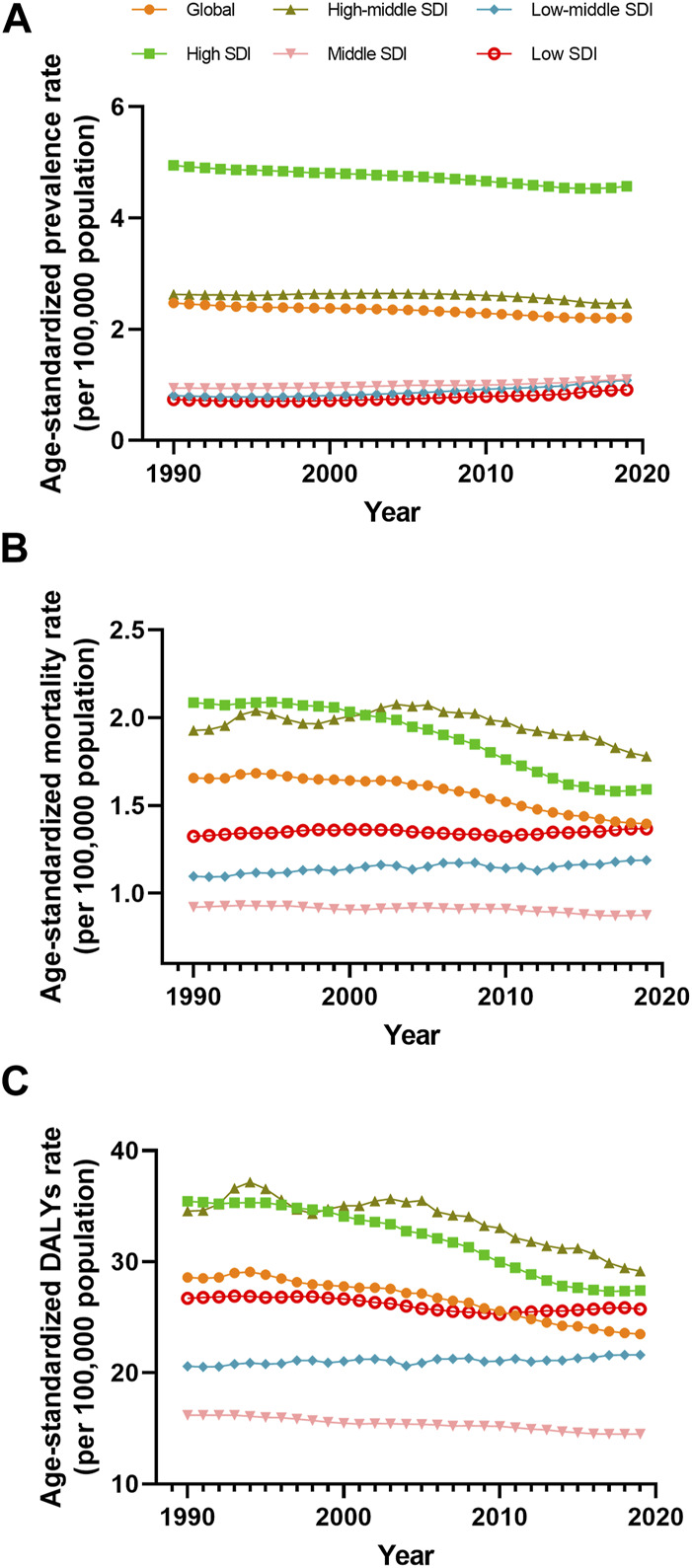
Prevalence **(A)**, mortality **(B)** and disability-adjusted life years **(C)** age-standardized rate of vascular intestinal disorders by sociodemographic index quintile, Global, from 1990 to 2019. DALYs, disability-adjusted life years; ASR, age-standardized rate; SDI, socio-demographic index.

The disease burden related to VID displayed distinct patterns across different SDI quintiles. A clear trend emerged, indicating that higher SDI levels were associated with higher prevalence ASR of VID from 1990 to 2019 (Figure 5A). Specifically, as compared to other areas, ASR prevalence is significantly higher in high SDI areas. The high-middle SDI regions showed prevalence ASR close to the global level but substantially higher than in middle, low-middle, and low SDI regions. Throughout this period, the prevalence ASR decreased in the high and high-middle SDI quintiles, while it increased in the medium, low-middle, and low SDI quintiles (Table 1). Regarding mortality ASR, the highest rates were observed in the high SDI region in the previous decade, with subsequently in the high-middle SDI region from 2001 to 2019. Conversely, the middle SDI quintile had the lowest mortality ASR from 1999 to 2019 (Figure 5B). The mortality ASR decreased in the high, high-middle, and middle SDI quintiles from 1990 to 2019, while it increased in the low-middle and low SDI quintiles (Table 1). As for the ASR of DALYs, high SDI quintiles exhibited the highest values in 1990, 1991, and 1998, whereas high-middle SDI quintiles had the highest values in all other years (Figure 5C). Between 1990 and 2019, the ASR of DALYs decreased for all SDI quintiles, except for the low-middle SDI quintile (Table 1).

Additionally, we explored the connections between SDI and the burden of VID in 2019. Positive correlations were evident between SDI and the prevalence ASR (R = 0.672, *p* < 0.001) as well as the mortality ASR (R = 0.196, *p* = 0.005) (Figures 3A, B). However, no significant correlation was found between SDI and the DALYs ASR (Figure 3C). To gain deeper insights into the changes in burden of the country-level over the 30-year period, stratified by SDI, we examined the relationship between SDI and the changes in mortality and DALYs ASR. SDI was negatively correlated (Supplementary Figures S2B, C) with both the changes in mortality ASR (R = −0.216, *p* = 0.002) and DALYs ASR (R = −0.175, *p* = 0.012). However, the SDI was not significantly associated with changes in prevalence ASR (Supplementary Figure S2A).

## Discussion

Our study represents a pioneering assessment of the burden of VID on a global, regional, and national scale, considering sex and age over a span of 30 years. There is a consistent increase in the prevalence of VID since 1990, with a recorded figure of 175,740 cases in 2019. However, we observed a decline in prevalence ASR by 10.5%, from 2.47 per 100,000 population in 1990 to 2.21 in 2019. This rise in prevalence, coupled with the decrease in ASR, can primarily be attributed to population growth and the global aging phenomenon experienced in the majority of countries between 1990 and 2019. Notably, VID frequently coexists with cardiovascular diseases, such as atherosclerosis [16], which leads to the narrowing and obstruction of arteries supplying blood to the intestines. Atrial fibrillation, a significant contributor to mesenteric thrombosis, is diagnosed in 47% of individuals with mesenteric ischemia [31]. Moreover, global prevalence rates of ischemic heart disease and atrial fibrillation have also shown an upward trend from 1990 to 2019(28), which may have contributed to the augmented prevalence of VID. Similar to ischemic heart disease, the development of VID is associated with diabetes [32] and alcohol use disorder [33, 34]. Furthermore, age-related alterations in colonic blood supply may account for the increasing prevalence of ischemic colitis observed with advancing age [35].

Death resulting from VID is commonly associated with complications such as intestinal perforation or uncontrolled infection [36]. Globally, the estimated number of deaths due to VID increased from 54,583.12 in 1990 to 106,575.78 in 2019, despite a substantial decrease in mortality ASR over the same period. This decline in mortality rates has persisted and is observed in most countries. One possible reason is the earlier and more effective management of VID patients, resulting in a reduced number of individuals experiencing worsening conditions. Advances in diagnostic techniques have played a significant role in preventing serious complications and lowering mortality rates. The relative availability of non-invasive imaging techniques such as computed tomography angiography and magnetic resonance angiograph has enabled more accurate and rapid diagnosis of VID without the need for invasive angiography [37]. The widespread adoption of endovascular interventions and surgical revascularization has also contributed to the effective and timely treatment of VID, leading to the prevention of more deaths [38–40]. In particular, the introduction of endovascular treatment has resulted in lower rates of bowel resection and postoperative mortality compared to traditional open surgery [41]. Notably, Eastern Europe exhibited the highest mortality ASR for VID in 2019 and the largest increase in mortality ASR from 1990 to 2019. However, there is a lack of definitive studies investigating this intriguing finding. Further research is warranted to understand the specific factors contributing to the elevated mortality rates in this region.

VID and its complications have a profound impact on individuals, significantly affecting their daily activities and diminishing their overall quality of life [7, 8]. Furthermore, the condition imposes substantial economic and social burdens [7, 9, 10]. One particularly noteworthy consequence of VID is short bowel syndrome resulting from bowel resection, which necessitates long-term home parenteral nutrition. This places considerable medical resource costs and financial distress on patients and their families, further compromising their quality of life [42]. Our study also revealed a total of 1,872,339 DALYs attributed to VID in 2019, despite a decreasing trend in the ASR of DALYs from 1990 to 2019. This decline in DALYs ASR, similar to mortality, may be attributed to advancements in treatment strategies. For example, in chronic mesenteric ischemia, where inadequate mesenteric circulation leads to persistent abdominal symptoms, patients’ quality of life can be significantly affected. A study by Blauw et al. assessed the quality of life before and after revascularization in chronic mesenteric ischemia using the EuroQol-5D (EQ-5D) questionnaire and found a significant improvement in quality of life (EQ-5D increased from 0.70 to 0.81, *p* = 0.02) [8]. However, further robust investigations are warranted to explore the factors contributing to the variation in DALYs ASR in VID.

We conducted an investigation into the burden of VID across different sex and age, and our findings revealed distinct patterns. Specifically, we observed a significant increase in the burden of VID with advancing age. These findings are consistent with previous research, which supports the idea that older age independently contributes to adverse outcomes in patients with acute mesenteric ischemia [43]. Additionally, the elderly age group has been associated with a higher risk of mortality in individuals with mesenteric vein thrombosis [44]. Furthermore, our study demonstrated that men generally exhibited higher prevalence ASR of VID across most regions, with exceptions in High-income North America, High-income Asia Pacific, and Southeast Asia. While a few mouse-based studies have explored sex differences in the mechanisms of ischemia/reperfusion injury in mesenteric vessels [45, 46], there remains a need for further investigations to fully understand the role of sex in the pathogenesis of VID using human-based studies.

SDI plays a crucial role as a social and economic indicator, reflecting the educational and income levels within a population. In our study, we discovered a positive correlation between the prevalence ASR of VID and SDI. Furthermore, when analyzing different SDI quintiles, we observed that the high and high-middle SDI regions exhibited the highest burden over the 30 year period. These findings suggest countries with higher levels of socio-economic development generally experience a greater burden of VID. Interestingly, we found that the prevalence of VID was considerably higher in high and high-middle SDI regions, and the resulting large population base might have masked the correlation between mortality and DALYs with SDI. Consequently, we conclude that higher SDI levels may be associated with a lower mortality and DALYs burden of VID in a given area. It is worth noting that a study demonstrated an increased risk of death from acute mesenteric ischemia among individuals residing in higher-income residential areas (OR = 1.049 [1.009–1.091], *p* = 0.017) [47]. However, there is still a lack of research exploring the fundamental relationship between VID burden and education or income. In this context, the Burkitt hypothesis proposes a link between diseases more prevalent in high-income countries, such as diabetes, atherosclerosis, and obesity, and lower dietary fiber consumption [48]. Additionally, human intervention studies have provided evidence of an association between VID-related diseases, including diabetes [49–51] and atherosclerosis [52], and low dietary fiber intake.

It is essential to acknowledge several limitations in our research. Firstly, the accuracy and robustness of GBD 2019 estimates, like all studies based on GBD research, may be influenced by potential biases associated with the import data. To mitigate these concerns, we utilized DisMod-MR 2.1, which partially addresses data-related biases. Nevertheless, it is important to note that despite combining YLL and YLD to calculate DALYs, some information gaps may still persist. Additionally, due to limited available data from a few countries and territories, our burden estimates for VID relied on modeled data rather than population-based data, introducing potential uncertainty in our findings. To enhance the accuracy and reliability of future studies, it is crucial to prioritize conducting comprehensive health surveys to gather representative data from a wider range of countries. Secondly, in GBD 2019, VID is defined as an amalgamation of mesenteric ischemia, ischemic colitis, and angiodysplasia of the intestine. While this approach offers a comprehensive overview of the burden of vascular defects in the intestine, it is important to investigate these diseases separately in future studies to assess their individual disease burden more precisely. Lastly, the GBD 2019 database lacks specific identification of risk factors for VID. Further health surveys are warranted to explore potential risk factors associated with VID, as understanding these factors can provide valuable insights for developing targeted prevention and management strategies in the future.

This study analyzed the VID burden across various dimensions, including time, region, sex, age, and SDI levels. Our findings revealed significant gender disparities in VID, with males exhibiting higher burden in most regions, except for the high SDI quintile regions. While gender differences are evident, further mechanistic studies are required to fully understand the underlying reasons for these variations. Additionally, our investigation identified correlations between VID and education and income levels. However, the specific factors contributing to these associations remain elusive, highlighting the need for additional research efforts to unravel the complex relationships influencing disease patterns. Furthermore, it is important to note that predictive research focused on the global burden of VID disease is still limited, which is crucial for guiding future prevention and treatment strategies.

### Conclusion

Our study revealed a consistent global decrease in the prevalence, mortality, and DALYs ASR for VID between 1990 and 2019. However, when comparing different SDI quintiles, the high and high-middle SDI quintiles exhibited the highest burden ASR during the same period. In addition, prevalence was generally higher in men, except for persons >75 years of age in 2019 and persons in areas with high SDI during the 30 year period. These findings emphasize the importance of implementing targeted measures to combat VID, particularly in countries with higher SDI levels and those specifically burdened by VID.
