# Supplementary material for: The Global Burden of Vascular Intestinal Disorders in 204 Countries and Territories From 1990 to 2019: Global Burden of Diseases Study
Source: Int J Public Health. 2023 Sep 26;68:1606297. doi: 10.3389/ijph.2023.1606297 (PMC10562586; doi:10.3389/ijph.2023.1606297)
Supplement: Supplementary file 1 [file DataSheet1.pdf]

International Journal of Public Health

Supplementary materials for the submission of manuscript titled "The Global Burden of Vascular Intestinal Disorders in 204 Countries and Territories From 1990 to 2019: Global Burden of Diseases Study"

Table of Contents

Figure S1: Prevalence (a) mortality (c) and DALYs (e) ASR in 2019 and percentage change in prevalence (b), mortality (d) and DALYs (f) ASR from 1990 to 2019 of VID by sex across 21 GBD regions.

Figure S2: Correlation of percentage of the prevalence (a), mortality (b) and DALYs (c) ASR of VID in 2019 with different SDI in 204 countries and territories.

Figure S3: Prevalence (a), mortality (b) and DALYs (c) rate of VID by age and sex in 2019 at the global level.

Table S1: List of ICD-10 codes about vascular intestinal disorders.

Table S2: 204 countries or territories and 21 regions in GBD 2019.

Table S3: Prevalence cases and age-standardized prevalence rate of vascular intestinal disorders in 1990 and 2019, and estimated annual percentage change in age-standardized prevalence rate from 1990 to 2019 in 204 countries and regions.

Table S4: Deaths and age-standardized mortality rate of vascular intestinal disorders in 1990 and 2019, and estimated annual percentage change in age-standardized mortality rate from 1990 to 2019 in 204 countries and regions.

Table S5: DALYs and age-standardized DALYs rate of vascular intestinal disorders in 1990 and 2019, and estimated annual percentage change in age-standardized DALYs rate from 1990 to 2019 in 204 countries and regions.

Supplementary materials 1: Input Data and Methodological Summary for VID by GBD 2019 Diseases and Injuries Collaborators (licensed under CC BY 4.0)

Figure S1 Prevalence (a) mortality (c) and DALYs (e) ASR in 2019 and percentage change in prevalence (b), mortality (d) and DALYs (f) ASR from 1990 to 2019 of VID by sex across 21 GBD regions.

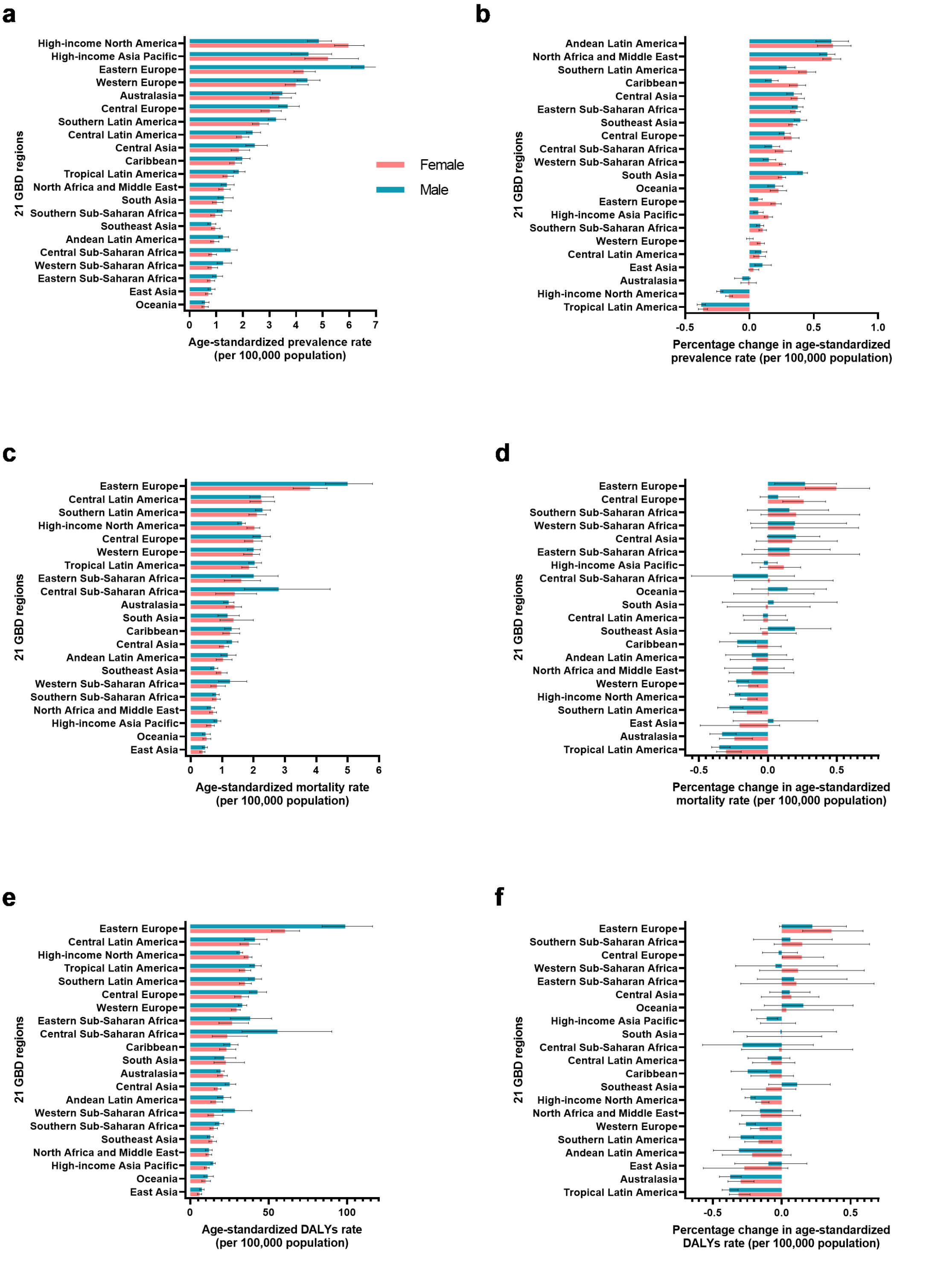

DALYs, disability-adjusted life-years; ASR, age-standardized rate; GBD, Global Burden of Diseases, Injuries, and Risk Factors Study.

Figure S2 Correlation of percentage of the prevalence (a), mortality (b) and DALYs (c) ASR of VID in 2019 with different SDI in 204 countries and territories.

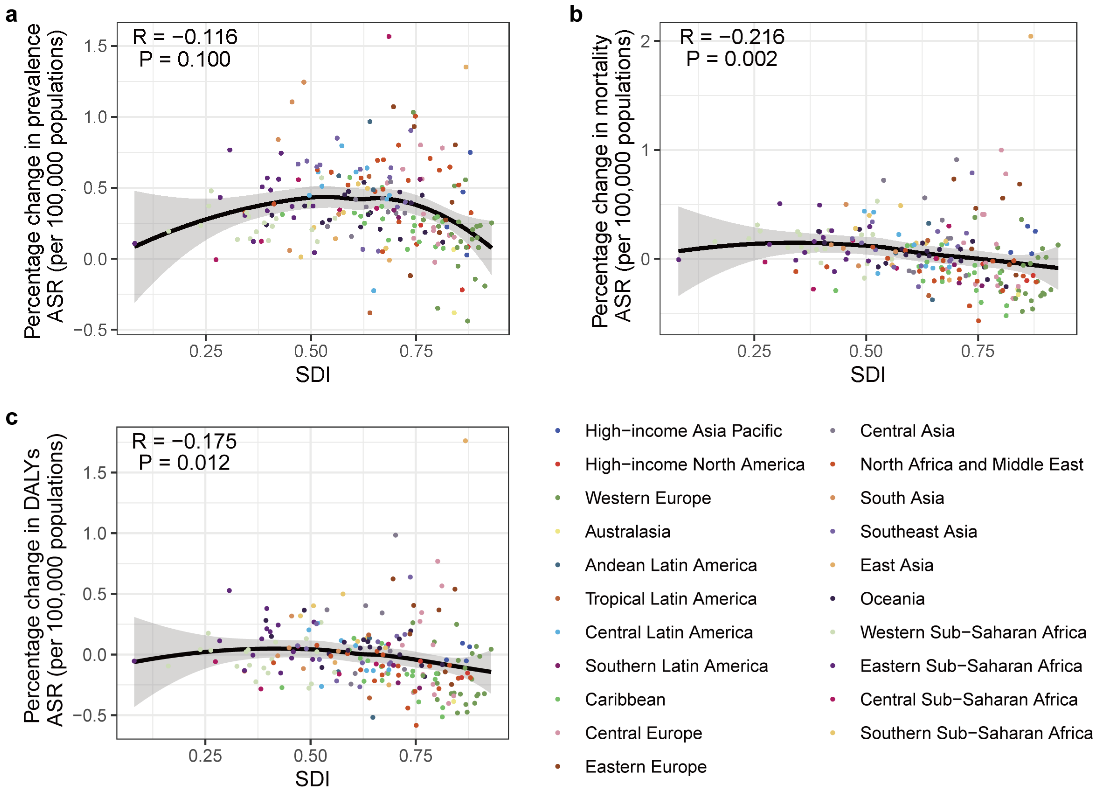

DALYs, disability-adjusted life years; ASR, age-standardized rate; SDI, socio-demographic index.

Figure S3 Prevalence (a), mortality (b) and DALYs (c) rate of VID by age and sex in 2019 at the global level.

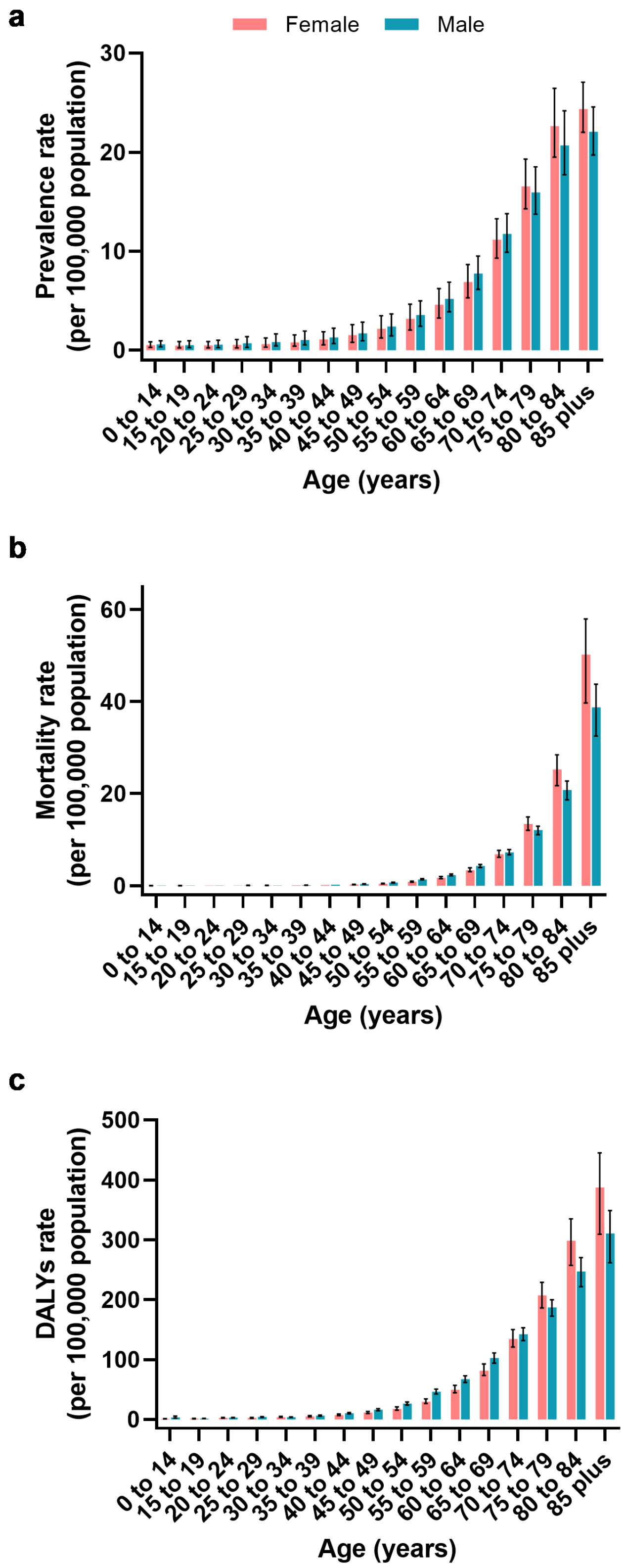

DALYs, disability-adjusted life years; VID, vascular intestinal disorders.

Table S1 List of ICD-10 codes about vascular intestinal disorders.

ICD-10	Diagnoses
**K55**	**Vascular disorders of intestine**
**K55.0**	**Acute vascular disorders of intestine**
	Acute:
	fulminant ischaemic colitis
	intestinal infarction
	small intestine ischaemia
	Mesenteric (artery)(vein):
	embolism
	infarction
	thrombosis
	Subacute ischaemic colitis
**K55.1**	**Chronic vascular disorders of intestine**
	Chronic ischaemic:
	colitis
	enteritis
	enterocolitis
	Ischaemic stricture of intestine
	Mesenteric:
	atherosclerosis
	vascular insufficiency
**K55.2**	**Angiodysplasia of colon**
**K55.3**	**Angiodysplasia of small intestine**
**K55.8**	**Other vascular disorders of intestine**
**K55.9**	**Vascular disorder of intestine, unspecified**

ICD=International Classification of Diseases.

Table S2 204 countries or territories and 21 regions in GBD 2019

Geographic Regions	Countries or territories
**East Asia**	China
	Democratic People's Republic of Korea
	Taiwan (Province of China)
**Southeast Asia**	Cambodia
	Indonesia
	Lao People's Democratic Republic
	Malaysia
	Maldives
	Mauritius
	Myanmar
	Philippines
	Seychelles
	Sri Lanka
	Thailand
	Timor-Leste
	Viet Nam
**Oceania**	American Samoa
	Cook Islands
	Fiji
	Guam
	Kiribati
	Marshall Islands
	Micronesia (Federated States of)
	Nauru
	Niue
	Northern Mariana Islands
	Palau
	Papua New Guinea
	Samoa
	Solomon Islands
	Tokelau
	Tonga
	Tuvalu
	Vanuatu
**Central Asia**	Armenia
	Azerbaijan
	Georgia
	Kazakhstan
	Kyrgyzstan
	Mongolia
	Tajikistan

	Turkmenistan
	Uzbekistan
**Central Europe**	Albania
	Bosnia and Herzegovina
	Bulgaria
	Croatia
	Czechia
	Hungary
	North Macedonia
	Montenegro
	Poland
	Romania
	Serbia
	Slovakia
	Slovenia
**Eastern Europe**	Belarus
	Estonia
	Latvia
	Lithuania
	Republic of Moldova
	Russian Federation
	Ukraine
**High-income Asia Pacific**	Brunei Darussalam
	Japan
	Republic of Korea
	Singapore
**Australasia**	Australia
	New Zealand
**Western Europe**	Andorra
	Austria
	Belgium
	Cyprus
	Denmark
	Finland
	France
	Germany
	Greece
	Iceland
	Ireland
	Israel
	Italy
	Luxembourg
	Malta

	Monaco
	Netherlands
	Norway
	Portugal
	San Marino
	Spain
	Sweden
	Switzerland
	United Kingdom
**Southern Latin America**	Argentina
	Chile
	Uruguay
**High-income North America**	Canada
	Greenland
	United States of America
**Caribbean**	Antigua and Barbuda
	Bahamas
	Barbados
	Belize
	Bermuda
	Cuba
	Dominica
	Dominican Republic
	Grenada
	Guyana
	Haiti
	Jamaica
	Puerto Rico
	Saint Kitts and Nevis
	Saint Lucia
	Saint Vincent and the Grenadines
	Suriname
	Trinidad and Tobago
	United States Virgin Islands
**Andean Latin America**	Bolivia (Plurinational State of)
	Ecuador
	Peru
**Central Latin America**	Colombia
	Costa Rica
	El Salvador
	Guatemala
	Honduras
	Mexico

	Nicaragua<br>Panama<br>Venezuela (Bolivarian Republic of)
**Tropical Latin America**	Brazil<br>Paraguay
**North Africa and Middle East**	Afghanistan<br>Algeria<br>Bahrain<br>Egypt<br>Iran (Islamic Republic of)<br>Iraq<br>Jordan<br>Kuwait<br>Lebanon<br>Libya<br>Morocco<br>Palestine<br>Oman<br>Qatar<br>Saudi Arabia<br>Sudan<br>Syrian Arab Republic<br>Tunisia<br>Turkey<br>United Arab Emirates<br>Yemen
**South Asia**	Bangladesh<br>Bhutan<br>India<br>Nepal<br>Pakistan
**Central Sub-Saharan Africa**	Angola<br>Central African Republic<br>Congo<br>Democratic Republic of the Congo<br>Equatorial Guinea<br>Gabon
**Eastern Sub-Saharan Africa**	Burundi<br>Comoros<br>Djibouti<br>Eritrea<br>Ethiopia<br>Kenya

	Madagascar
	Malawi
	Mozambique
	Rwanda
	Somalia
	South Sudan
	United Republic of Tanzania
	Uganda
	Zambia
**Southern Sub-Saharan Africa**	Botswana
	Lesotho
	Namibia
	South Africa
	Eswatini
	Zimbabwe
**Western Sub-Saharan Africa**	Benin
	Burkina Faso
	Cameroon
	Cabo Verde
	Chad
	Côte d'Ivoire
	Gambia
	Ghana
	Guinea
	Guinea-Bissau
	Liberia
	Mali
	Mauritania
	Niger
	Nigeria
	Sao Tome and Principe
	Senegal
	Sierra Leone
	Togo

GBD, Global Burden of Disease, Injuries, and Risk Factors Study.

Table S3 Prevalence cases and age-standardized prevalence rate of vascular intestinal disorders in 1990 and 2019, and estimated annual percentage change in age-standardized prevalence rate from 1990 to 2019 in 204 countries and regions.

Measure	1990		2019		1990-2019
	Cases (95% UI)	ASR (95% UI)	Cases (95% UI)	ASR (95% UI)	EAPC (95% UI)
Afghanistan	49 (39 to 64)	0.53 (0.44 to 0.66)	171 (132 to 230)	0.7 (0.58 to 0.87)	1.14% (0.84 to 1.43)
Albania	36 (29 to 48)	1.24 (1.01 to 1.57)	65 (54 to 78)	1.91 (1.58 to 2.38)	1.65% (1.55 to 1.75)
Algeria	161 (132 to 206)	0.93 (0.79 to 1.12)	503 (418 to 627)	1.37 (1.16 to 1.67)	1.27% (1.19 to 1.35)
American Samoa	0 (0 to 0)	0.76 (0.64 to 0.94)	0 (0 to 1)	0.87 (0.73 to 1.07)	0.48% (0.44 to 0.53)
Andorra	2 (2 to 2)	3.54 (3.13 to 3.98)	6 (5 to 7)	4.35 (3.8 to 4.92)	0.68% (0.5 to 0.87)
Angola	56 (42 to 77)	0.7 (0.58 to 0.87)	235 (182 to 312)	1.09 (0.93 to 1.32)	1.71% (1.51 to 1.92)
Antigua and Barbuda	1 (0 to 1)	0.98 (0.81 to 1.22)	1 (1 to 2)	1.27 (1.04 to 1.57)	0.87% (0.81 to 0.93)
Argentina	610 (552 to 681)	1.89 (1.71 to 2.11)	1294 (1168 to 1442)	2.44 (2.19 to 2.73)	0.64% (0.49 to 0.79)
Armenia	77 (68 to 90)	2.7 (2.4 to 3.06)	158 (144 to 174)	4.16 (3.76 to 4.65)	1.83% (1.71 to 1.95)
Australia	643 (585 to 716)	3.31 (3 to 3.69)	1421 (1285 to 1587)	3.53 (3.15 to 3.99)	0.11% (-0.02 to 0.24)
Austria	377 (346 to 411)	3.11 (2.84 to 3.4)	488 (442 to 546)	2.95 (2.64 to 3.36)	-0.38% (-0.67 to -0.09)
Azerbaijan	90 (70 to 119)	1.28 (1.04 to 1.65)	167 (135 to 212)	1.71 (1.4 to 2.16)	1.31% (1.05 to 1.58)
Bahamas	3 (3 to 4)	1.72 (1.51 to 1.99)	7 (6 to 8)	1.86 (1.62 to 2.16)	0.17% (0.1 to 0.23)
Bahrain	5 (4 to 7)	2.53 (2.28 to 2.82)	27 (22 to 34)	2.79 (2.44 to 3.17)	-0.07% (-0.41 to 0.28)
Bangladesh	474 (371 to 634)	0.6 (0.5 to 0.78)	1895 (1623 to 2278)	1.36 (1.17 to 1.59)	3.08% (2.97 to 3.19)
Barbados	3 (3 to 4)	1.09 (0.93 to 1.35)	5 (4 to 6)	1.31 (1.1 to 1.6)	0.53% (0.5 to 0.57)
Belarus	334 (297 to 380)	2.74 (2.43 to 3.11)	804 (727 to 885)	5.29 (4.77 to 5.84)	2.79% (2.55 to 3.03)
Belgium	717 (663 to 778)	4.79 (4.4 to 5.24)	1318 (1220 to 1418)	6.04 (5.51 to 6.57)	0.59% (0.45 to 0.73)
Belize	1 (1 to 2)	0.76 (0.62 to 0.95)	3 (3 to 4)	1 (0.83 to 1.24)	0.92% (0.87 to 0.98)
Benin	32 (23 to 44)	0.67 (0.53 to 0.86)	94 (69 to 129)	0.86 (0.71 to 1.09)	0.84% (0.76 to 0.91)
Bermuda	1 (1 to 1)	1.71 (1.48 to 1.99)	2 (2 to 3)	2.16 (1.86 to 2.53)	0.71% (0.6 to 0.81)
Bhutan	3 (2 to 4)	0.73 (0.61 to 0.9)	10 (8 to 12)	1.54 (1.32 to 1.82)	2.88% (2.78 to 2.98)
Bolivia (Plurinational State of)	26 (21 to 34)	0.57 (0.49 to 0.69)	88 (74 to 107)	0.9 (0.78 to 1.07)	1.45% (1.38 to 1.51)
Bosnia and Herzegovina	58 (49 to 72)	1.41 (1.2 to 1.7)	108 (95 to 126)	2.3 (1.99 to 2.76)	2.2% (1.97 to 2.43)
Botswana	9 (7 to 12)	0.85 (0.7 to 1.06)	25 (20 to 31)	1.27 (1.07 to 1.52)	1.32% (1.23 to 1.41)
Brazil	2744 (2373 to 3289)	2.61 (2.33 to 2.96)	3756 (3337 to 4308)	1.61 (1.44 to 1.84)	-1.43% (-1.63 to -1.23)
Brunei Darussalam	7 (5 to 9)	3.54 (2.99 to 4.29)	17 (14 to 21)	4.34 (3.71 to 5.13)	0.73% (0.66 to 0.81)
Bulgaria	195 (170 to 229)	1.86 (1.6 to 2.22)	339 (310 to 375)	2.9 (2.55 to 3.34)	1.99% (1.81 to 2.17)
Burkina Faso	52 (37 to 74)	0.56 (0.44 to 0.73)	149 (110 to 206)	0.76 (0.62 to 0.97)	1.15% (1.07 to 1.23)
Burundi	24 (18 to 33)	0.53 (0.44 to 0.68)	61 (47 to 81)	0.76 (0.65 to 0.93)	1.53% (1.37 to 1.69)
Cabo Verde	3 (2 to 3)	0.77 (0.62 to 0.97)	6 (5 to 8)	1.2 (0.99 to 1.51)	1.67% (1.61 to 1.74)
Cambodia	29 (23 to 39)	0.42 (0.36 to 0.52)	92 (75 to 115)	0.7 (0.6 to 0.84)	1.91% (1.78 to 2.03)
Cameroon	67 (49 to 93)	0.69 (0.55 to 0.88)	221 (168 to 297)	0.94 (0.78 to 1.18)	1.13% (1 to 1.26)
Canada	1847 (1665 to 2082)	5.83 (5.23 to 6.58)	3923 (3581 to 4323)	6.32 (5.69 to 7.13)	0.47% (0.4 to 0.55)
Central African Republic	15 (11 to 19)	0.73 (0.62 to 0.88)	28 (22 to 38)	0.73 (0.62 to 0.89)	0.05% (0 to 0.11)
Chad	39 (29 to 53)	0.7 (0.57 to 0.88)	121 (87 to 166)	0.86 (0.71 to 1.08)	0.66% (0.58 to 0.74)
Chile	224 (202 to 250)	2.21 (2.02 to 2.45)	813 (736 to 904)	3.45 (3.12 to 3.85)	1.49% (1.39 to 1.59)
China	6287 (5099 to 7843)	0.69 (0.58 to 0.84)	12906 (10637 to 15802)	0.69 (0.58 to 0.82)	-0.12% (-0.22 to -0.02)

Measure	1990		2019		1990-2019
	Cases (95% UI)	ASR (95% UI)	Cases (95% UI)	ASR (95% UI)	EAPC (95% UI)
Colombia	356 (304 to 428)	1.65 (1.47 to 1.88)	1387 (1245 to 1570)	2.71 (2.43 to 3.06)	1.58% (1.4 to 1.75)
Comoros	3 (2 to 4)	0.71 (0.6 to 0.87)	6 (5 to 7)	0.97 (0.82 to 1.16)	1.25% (1.16 to 1.34)
Congo	16 (12 to 21)	0.85 (0.72 to 1.03)	45 (36 to 59)	1.14 (0.97 to 1.37)	1.22% (1.06 to 1.38)
Cook Islands	0 (0 to 0)	0.75 (0.62 to 0.93)	0 (0 to 0)	1.08 (0.92 to 1.31)	1.23% (1.19 to 1.27)
Costa Rica	56 (50 to 65)	2.94 (2.68 to 3.25)	207 (188 to 228)	4.19 (3.81 to 4.62)	1.31% (1.12 to 1.5)
Côte d'Ivoire	89 (64 to 123)	0.78 (0.62 to 1)	214 (162 to 290)	0.96 (0.79 to 1.21)	0.63% (0.52 to 0.74)
Croatia	240 (219 to 263)	4.01 (3.65 to 4.45)	320 (295 to 348)	3.92 (3.55 to 4.39)	-0.17% (-0.23 to -0.1)
Cuba	219 (195 to 248)	2.11 (1.89 to 2.39)	510 (463 to 565)	2.89 (2.6 to 3.22)	1.17% (1.12 to 1.22)
Cyprus	12 (10 to 14)	1.5 (1.3 to 1.73)	43 (38 to 50)	2.27 (1.99 to 2.61)	1.5% (1.38 to 1.62)
Czechia	327 (293 to 370)	2.61 (2.31 to 3.03)	513 (458 to 577)	3.05 (2.66 to 3.54)	-0.04% (-0.2 to 0.12)
Democratic People's Republic of Korea	128 (103 to 162)	0.72 (0.59 to 0.88)	249 (203 to 305)	0.82 (0.68 to 0.99)	0.52% (0.28 to 0.77)
Democratic Republic of the Congo	268 (212 to 347)	1.06 (0.92 to 1.25)	656 (530 to 830)	1.19 (1.04 to 1.38)	0.26% (0.07 to 0.46)
Denmark	276 (252 to 303)	3.46 (3.14 to 3.82)	323 (288 to 359)	2.96 (2.62 to 3.35)	-0.7% (-0.94 to -0.46)
Djibouti	3 (2 to 4)	0.76 (0.63 to 0.94)	9 (7 to 11)	0.98 (0.82 to 1.18)	1.04% (0.86 to 1.21)
Dominica	1 (1 to 1)	1.43 (1.26 to 1.65)	1 (1 to 1)	1.53 (1.32 to 1.78)	0.09% (-0.04 to 0.22)
Dominican Republic	44 (35 to 59)	0.74 (0.61 to 0.94)	118 (98 to 148)	1.17 (0.98 to 1.44)	1.51% (1.46 to 1.56)
Ecuador	51 (42 to 64)	0.72 (0.63 to 0.85)	215 (191 to 243)	1.42 (1.27 to 1.59)	2.51% (2.44 to 2.58)
Egypt	300 (240 to 397)	0.7 (0.58 to 0.89)	932 (752 to 1189)	1.19 (0.99 to 1.45)	1.75% (1.65 to 1.85)
El Salvador	35 (28 to 45)	0.83 (0.7 to 1.02)	89 (76 to 106)	1.49 (1.27 to 1.77)	2.11% (2.01 to 2.21)
Equatorial Guinea	2 (2 to 3)	0.69 (0.59 to 0.84)	16 (12 to 21)	1.78 (1.54 to 2.1)	3.94% (3.6 to 4.27)
Eritrea	12 (9 to 17)	0.52 (0.43 to 0.65)	39 (30 to 51)	0.82 (0.7 to 1)	1.48% (1.4 to 1.57)
Estonia	96 (88 to 105)	4.83 (4.43 to 5.3)	177 (163 to 192)	6.75 (6.15 to 7.37)	1.61% (1.35 to 1.88)
Eswatini	5 (3 to 7)	0.68 (0.54 to 0.87)	9 (7 to 12)	0.9 (0.74 to 1.13)	0.89% (0.64 to 1.13)
Ethiopia	224 (165 to 314)	0.55 (0.45 to 0.69)	606 (448 to 820)	0.72 (0.59 to 0.9)	1.05% (0.87 to 1.22)
Fiji	4 (3 to 5)	0.63 (0.53 to 0.78)	6 (5 to 8)	0.76 (0.64 to 0.94)	0.52% (0.47 to 0.58)
Finland	228 (206 to 255)	3.21 (2.89 to 3.62)	428 (385 to 475)	3.6 (3.19 to 4.09)	0.25% (0.17 to 0.32)
France	2967 (2743 to 3228)	3.56 (3.27 to 3.89)	5388 (4923 to 5897)	4.17 (3.75 to 4.65)	0.55% (0.46 to 0.64)
Gabon	9 (7 to 11)	1.05 (0.89 to 1.28)	20 (16 to 25)	1.43 (1.22 to 1.71)	1.04% (0.93 to 1.15)
Gambia	7 (5 to 10)	0.8 (0.66 to 1.01)	17 (13 to 23)	0.91 (0.75 to 1.15)	0.26% (0.2 to 0.31)
Georgia	77 (63 to 98)	1.38 (1.12 to 1.76)	85 (75 to 97)	1.83 (1.56 to 2.2)	1.26% (1.02 to 1.5)
Germany	5728 (5250 to 6263)	4.67 (4.27 to 5.14)	8680 (8004 to 9532)	5.04 (4.55 to 5.63)	-0.09% (-0.33 to 0.15)
Ghana	111 (81 to 151)	0.78 (0.63 to 1)	279 (212 to 372)	1 (0.81 to 1.27)	0.46% (0.3 to 0.62)
Greece	302 (262 to 352)	2.09 (1.8 to 2.46)	586 (525 to 657)	2.68 (2.34 to 3.13)	0.86% (0.83 to 0.89)
Greenland	2 (2 to 2)	4.72 (4.24 to 5.26)	4 (3 to 4)	5.84 (5.26 to 6.51)	0.83% (0.75 to 0.91)
Grenada	1 (1 to 1)	0.82 (0.68 to 1.03)	1 (1 to 2)	1.24 (1.04 to 1.52)	1.31% (1.25 to 1.37)
Guam	1 (1 to 1)	0.97 (0.82 to 1.18)	2 (2 to 2)	1.09 (0.91 to 1.32)	0.25% (0.2 to 0.31)
Guatemala	46 (35 to 61)	0.69 (0.57 to 0.87)	147 (120 to 189)	1.04 (0.87 to 1.3)	1.47% (1.38 to 1.55)
Guinea	45 (34 to 61)	0.71 (0.57 to 0.91)	93 (69 to 125)	0.81 (0.65 to 1.04)	0.25% (0.16 to 0.34)
Guinea-Bissau	7 (5 to 9)	0.68 (0.55 to 0.87)	13 (9 to 18)	0.78 (0.63 to 0.99)	0.28% (0.23 to 0.33)
Guyana	5 (4 to 6)	0.85 (0.73 to 1.02)	8 (6 to 9)	1.1 (0.94 to 1.32)	0.77% (0.67 to 0.88)

Measure	1990		2019		1990-2019
	Cases (95% UI)	ASR (95% UI)	Cases (95% UI)	ASR (95% UI)	EAPC (95% UI)
Haiti	30 (23 to 40)	0.58 (0.49 to 0.72)	73 (59 to 95)	0.76 (0.64 to 0.93)	0.97% (0.9 to 1.03)
Honduras	38 (31 to 48)	1.26 (1.11 to 1.46)	126 (110 to 150)	1.83 (1.63 to 2.09)	1.16% (1.11 to 1.21)
Hungary	511 (468 to 554)	3.8 (3.43 to 4.21)	770 (711 to 836)	4.55 (4.12 to 5.11)	0.33% (0.17 to 0.5)
Iceland	11 (10 to 13)	4.04 (3.59 to 4.59)	21 (19 to 24)	4.37 (3.8 to 4.99)	0.27% (0.11 to 0.43)
India	6169 (4833 to 8250)	0.88 (0.72 to 1.12)	15035 (12261 to 19469)	1.15 (0.96 to 1.46)	1.07% (0.94 to 1.21)
Indonesia	929 (757 to 1194)	0.74 (0.64 to 0.88)	1754 (1429 to 2227)	0.78 (0.66 to 0.95)	0.07% (-0.01 to 0.15)
Iran (Islamic Republic of)	418 (336 to 539)	1.11 (0.93 to 1.33)	918 (745 to 1152)	1.16 (0.97 to 1.42)	-0.25% (-0.39 to -0.11)
Iraq	102 (81 to 136)	0.8 (0.65 to 1.01)	375 (299 to 477)	1.19 (0.97 to 1.46)	1.65% (1.41 to 1.88)
Ireland	114 (103 to 126)	2.75 (2.47 to 3.08)	312 (283 to 346)	4.29 (3.84 to 4.76)	1.81% (1.68 to 1.93)
Israel	140 (127 to 157)	2.82 (2.55 to 3.16)	395 (359 to 442)	3.47 (3.12 to 3.93)	0.67% (0.58 to 0.75)
Italy	3656 (3335 to 4025)	4.15 (3.75 to 4.64)	3883 (3530 to 4287)	2.71 (2.43 to 3.05)	-1.85% (-1.98 to -1.72)
Jamaica	20 (17 to 25)	0.98 (0.83 to 1.2)	32 (27 to 40)	1.11 (0.92 to 1.39)	0.38% (0.33 to 0.42)
Japan	7290 (6114 to 8752)	4.69 (3.95 to 5.7)	11392 (9975 to 12992)	4.82 (4.04 to 5.83)	0.1% (-0.02 to 0.23)
Jordan	18 (15 to 23)	1 (0.87 to 1.16)	121 (102 to 147)	1.55 (1.34 to 1.8)	1.49% (1.39 to 1.6)
Kazakhstan	301 (252 to 367)	2.1 (1.8 to 2.49)	484 (416 to 570)	2.78 (2.4 to 3.25)	1.33% (1.17 to 1.49)
Kenya	161 (124 to 213)	1.06 (0.91 to 1.27)	394 (311 to 515)	1.11 (0.94 to 1.34)	0.05% (-0.08 to 0.17)
Kiribati	0 (0 to 0)	0.68 (0.59 to 0.81)	1 (1 to 1)	0.86 (0.75 to 1)	0.75% (0.71 to 0.79)
Kuwait	17 (13 to 22)	1.68 (1.46 to 1.97)	79 (64 to 99)	2.56 (2.23 to 2.95)	1.55% (1.43 to 1.68)
Kyrgyzstan	53 (41 to 69)	1.24 (1.01 to 1.56)	87 (69 to 114)	1.43 (1.17 to 1.82)	0.59% (0.41 to 0.78)
Lao People's Democratic Republic	12 (10 to 17)	0.39 (0.32 to 0.5)	36 (29 to 46)	0.66 (0.56 to 0.81)	1.95% (1.79 to 2.1)
Latvia	170 (156 to 185)	4.91 (4.49 to 5.41)	247 (229 to 267)	6.26 (5.74 to 6.82)	1.06% (0.93 to 1.19)
Lebanon	26 (21 to 32)	0.95 (0.8 to 1.18)	90 (77 to 107)	1.72 (1.46 to 2.04)	2.21% (2.16 to 2.26)
Lesotho	9 (7 to 12)	0.58 (0.48 to 0.74)	13 (10 to 17)	0.71 (0.58 to 0.89)	0.54% (0.34 to 0.74)
Liberia	13 (9 to 18)	0.63 (0.5 to 0.83)	31 (23 to 42)	0.76 (0.62 to 0.97)	0.85% (0.68 to 1.03)
Libya	28 (23 to 37)	0.98 (0.82 to 1.2)	73 (58 to 94)	1.23 (1.03 to 1.5)	0.94% (0.84 to 1.04)
Lithuania	149 (136 to 166)	3.48 (3.15 to 3.89)	361 (335 to 391)	6.27 (5.79 to 6.81)	2.51% (2.34 to 2.68)
Luxembourg	25 (23 to 27)	4.64 (4.24 to 5.1)	51 (47 to 56)	5.33 (4.8 to 5.91)	0.06% (-0.23 to 0.36)
Madagascar	57 (42 to 78)	0.57 (0.47 to 0.72)	149 (115 to 198)	0.77 (0.65 to 0.94)	1.05% (0.94 to 1.17)
Malawi	44 (34 to 60)	0.63 (0.52 to 0.77)	115 (90 to 149)	0.94 (0.81 to 1.12)	1.62% (1.47 to 1.78)
Malaysia	106 (86 to 136)	0.86 (0.74 to 1.03)	434 (378 to 505)	1.64 (1.45 to 1.88)	2.36% (2.21 to 2.51)
Maldives	1 (1 to 1)	0.54 (0.45 to 0.69)	4 (3 to 5)	1.01 (0.86 to 1.21)	2.34% (2.26 to 2.42)
Mali	54 (39 to 75)	0.61 (0.48 to 0.8)	169 (125 to 227)	0.91 (0.75 to 1.13)	1.42% (1.33 to 1.51)
Malta	9 (8 to 10)	2.12 (1.9 to 2.37)	25 (22 to 28)	2.75 (2.42 to 3.15)	0.65% (0.47 to 0.82)
Marshall Islands	0 (0 to 0)	0.54 (0.45 to 0.66)	0 (0 to 0)	0.69 (0.58 to 0.84)	0.85% (0.76 to 0.94)
Mauritania	14 (10 to 19)	0.72 (0.58 to 0.93)	35 (27 to 47)	1 (0.83 to 1.26)	1.08% (1.02 to 1.15)
Mauritius	6 (5 to 8)	0.67 (0.56 to 0.84)	14 (12 to 17)	0.92 (0.76 to 1.14)	0.93% (0.87 to 0.99)
Mexico	1298 (1090 to 1593)	2.45 (2.18 to 2.79)	2203 (1974 to 2505)	1.9 (1.72 to 2.15)	-1.16% (-1.27 to -1.05)
Micronesia (Federated States of)	0 (0 to 1)	0.53 (0.44 to 0.65)	1 (1 to 1)	0.76 (0.65 to 0.91)	1.26% (1.17 to 1.36)
Monaco	2 (2 to 2)	3.25 (2.9 to 3.68)	3 (3 to 4)	4.01 (3.58 to 4.57)	0.77% (0.72 to 0.82)
Mongolia	24 (18 to 33)	1.13 (0.91 to 1.46)	51 (40 to 68)	1.61 (1.3 to 2.05)	1.51% (1.33 to 1.68)

Measure	1990		2019		1990-2019
	Cases (95% UI)	ASR (95% UI)	Cases (95% UI)	ASR (95% UI)	EAPC (95% UI)
Montenegro	35 (32 to 38)	5.61 (5.13 to 6.17)	63 (58 to 69)	6.84 (6.24 to 7.53)	0.97% (0.83 to 1.1)
Morocco	137 (109 to 179)	0.72 (0.59 to 0.9)	385 (312 to 479)	1.15 (0.95 to 1.41)	1.6% (1.47 to 1.74)
Mozambique	60 (46 to 80)	0.61 (0.52 to 0.75)	203 (161 to 264)	1.08 (0.94 to 1.26)	2.09% (1.99 to 2.2)
Myanmar	120 (95 to 159)	0.38 (0.31 to 0.47)	310 (250 to 393)	0.62 (0.51 to 0.77)	1.93% (1.77 to 2.09)
Namibia	10 (7 to 13)	0.79 (0.65 to 0.99)	24 (19 to 30)	1.2 (1.02 to 1.46)	1.57% (1.39 to 1.75)
Nauru	0 (0 to 0)	0.71 (0.59 to 0.89)	0 (0 to 0)	0.87 (0.73 to 1.04)	0.54% (0.21 to 0.87)
Nepal	73 (59 to 95)	0.55 (0.46 to 0.67)	244 (206 to 301)	1.01 (0.87 to 1.21)	2.21% (2.11 to 2.3)
Netherlands	729 (663 to 798)	3.69 (3.34 to 4.07)	1358 (1233 to 1502)	4.2 (3.78 to 4.71)	0.51% (0.35 to 0.67)
New Zealand	188 (171 to 207)	4.73 (4.27 to 5.24)	218 (196 to 242)	2.93 (2.62 to 3.31)	-1.66% (-1.94 to -1.37)
Nicaragua	23 (17 to 30)	0.78 (0.65 to 0.97)	66 (55 to 82)	1.26 (1.07 to 1.53)	1.9% (1.76 to 2.03)
Niger	57 (41 to 80)	0.71 (0.57 to 0.9)	161 (115 to 222)	0.84 (0.7 to 1.05)	0.54% (0.44 to 0.63)
Nigeria	889 (671 to 1180)	1.06 (0.86 to 1.33)	2188 (1632 to 2934)	1.19 (0.98 to 1.48)	0.37% (0.34 to 0.4)
Niue	0 (0 to 0)	0.67 (0.55 to 0.82)	0 (0 to 0)	0.98 (0.83 to 1.18)	1.47% (1.39 to 1.55)
North Macedonia	42 (36 to 49)	2.17 (1.9 to 2.53)	68 (59 to 79)	2.49 (2.15 to 2.93)	0.32% (0.21 to 0.43)
Northern Mariana Islands	0 (0 to 0)	1.41 (1.25 to 1.63)	1 (1 to 1)	1.68 (1.47 to 1.93)	0.32% (0.14 to 0.49)
Norway	265 (241 to 293)	3.97 (3.54 to 4.46)	278 (246 to 314)	3.21 (2.8 to 3.7)	-1.22% (-1.38 to -1.07)
Oman	14 (11 to 18)	1.09 (0.92 to 1.34)	52 (41 to 71)	1.87 (1.59 to 2.22)	1.65% (1.55 to 1.76)
Pakistan	782 (634 to 1010)	0.96 (0.82 to 1.17)	1689 (1345 to 2205)	1.02 (0.87 to 1.24)	0.34% (0.2 to 0.48)
Palau	0 (0 to 0)	0.74 (0.62 to 0.91)	0 (0 to 0)	1.04 (0.88 to 1.24)	1.1% (0.99 to 1.2)
Palestine	11 (9 to 14)	0.86 (0.73 to 1.04)	42 (34 to 53)	1.31 (1.11 to 1.57)	1.29% (1.11 to 1.47)
Panama	26 (22 to 31)	1.44 (1.25 to 1.68)	86 (75 to 100)	2.09 (1.82 to 2.42)	1.02% (0.91 to 1.12)
Papua New Guinea	13 (10 to 18)	0.38 (0.29 to 0.5)	39 (30 to 53)	0.47 (0.37 to 0.61)	0.62% (0.5 to 0.74)
Paraguay	32 (27 to 40)	1.12 (0.97 to 1.34)	104 (90 to 123)	1.78 (1.56 to 2.08)	1.62% (1.55 to 1.68)
Peru	109 (87 to 141)	0.66 (0.55 to 0.8)	317 (266 to 391)	0.97 (0.82 to 1.19)	1.34% (1.27 to 1.41)
Philippines	309 (243 to 416)	0.64 (0.52 to 0.82)	673 (534 to 885)	0.69 (0.56 to 0.87)	0.26% (0.16 to 0.35)
Poland	859 (742 to 1023)	2.1 (1.79 to 2.52)	1358 (1236 to 1495)	2.32 (2.08 to 2.61)	0.01% (-0.47 to 0.5)
Portugal	327 (296 to 364)	2.43 (2.19 to 2.73)	1182 (1096 to 1280)	4.94 (4.52 to 5.4)	2.57% (2.27 to 2.88)
Puerto Rico	57 (50 to 66)	1.57 (1.37 to 1.83)	92 (80 to 108)	1.68 (1.41 to 2.03)	0.12% (0.01 to 0.23)
Qatar	8 (6 to 11)	2.37 (1.99 to 2.84)	82 (63 to 110)	3.9 (3.32 to 4.61)	1.96% (1.77 to 2.15)
Republic of Korea	1117 (876 to 1466)	2.79 (2.23 to 3.5)	3818 (3175 to 4604)	4.89 (4.11 to 5.88)	2.03% (1.97 to 2.08)
Republic of Moldova	98 (87 to 113)	2.2 (1.95 to 2.52)	249 (230 to 270)	4.56 (4.19 to 4.98)	3.23% (2.97 to 3.49)
Romania	656 (577 to 758)	2.57 (2.23 to 3.01)	1402 (1292 to 1526)	4.64 (4.17 to 5.24)	1.98% (1.89 to 2.08)
Russian Federation	8007 (7290 to 8846)	4.64 (4.21 to 5.16)	11978 (11214 to 12867)	5.5 (5.08 to 6.01)	0.86% (0.76 to 0.97)
Rwanda	31 (23 to 42)	0.59 (0.5 to 0.72)	86 (70 to 111)	1.03 (0.88 to 1.21)	2.4% (2.15 to 2.64)
Saint Kitts and Nevis	1 (1 to 1)	1.8 (1.59 to 2.07)	1 (1 to 2)	2.23 (1.96 to 2.57)	0.49% (0.39 to 0.59)
Saint Lucia	1 (1 to 2)	1.13 (0.97 to 1.33)	3 (2 to 3)	1.44 (1.24 to 1.71)	0.73% (0.68 to 0.78)
Saint Vincent and the Grenadines	1 (1 to 1)	0.78 (0.63 to 0.99)	1 (1 to 2)	0.97 (0.79 to 1.22)	0.75% (0.67 to 0.84)
Samoa	1 (1 to 1)	0.58 (0.48 to 0.71)	1 (1 to 2)	0.76 (0.64 to 0.93)	0.9% (0.79 to 1.02)
San Marino	1 (0 to 1)	1.81 (1.51 to 2.21)	1 (1 to 1)	2.18 (1.8 to 2.69)	0.73% (0.67 to 0.79)
Sao Tome and Principe	1 (1 to 1)	0.79 (0.64 to 1.01)	2 (1 to 2)	0.95 (0.78 to 1.22)	0.53% (0.47 to 0.59)
Saudi Arabia	107 (86 to 143)	1.03 (0.87 to 1.25)	432 (339 to 571)	1.67 (1.4 to 2)	1.65% (1.54 to 1.75)

Measure	1990		2019		1990-2019
	Cases (95% UI)	ASR (95% UI)	Cases (95% UI)	ASR (95% UI)	EAPC (95% UI)
Senegal	60 (44 to 81)	0.82 (0.67 to 1.03)	131 (103 to 172)	1.03 (0.87 to 1.27)	0.66% (0.6 to 0.72)
Serbia	330 (298 to 369)	3.11 (2.8 to 3.5)	592 (542 to 645)	4.09 (3.71 to 4.57)	0.9% (0.76 to 1.03)
Seychelles	0 (0 to 1)	0.76 (0.64 to 0.94)	1 (1 to 1)	1.06 (0.9 to 1.29)	1.12% (1.05 to 1.2)
Sierra Leone	24 (17 to 32)	0.64 (0.5 to 0.84)	60 (45 to 81)	0.85 (0.7 to 1.08)	1.04% (0.84 to 1.23)
Singapore	107 (85 to 138)	3.7 (3.04 to 4.55)	401 (339 to 482)	5.44 (4.65 to 6.47)	1.59% (1.5 to 1.69)
Slovakia	81 (68 to 97)	1.44 (1.2 to 1.75)	202 (178 to 229)	2.56 (2.23 to 2.98)	1.94% (1.7 to 2.18)
Slovenia	104 (95 to 114)	4.49 (4.07 to 5)	204 (186 to 225)	5.18 (4.67 to 5.83)	0.35% (0.3 to 0.41)
Solomon Islands	1 (1 to 1)	0.4 (0.33 to 0.52)	3 (2 to 4)	0.53 (0.43 to 0.65)	0.76% (0.65 to 0.87)
Somalia	25 (18 to 35)	0.46 (0.38 to 0.57)	72 (53 to 100)	0.51 (0.43 to 0.63)	0.52% (0.41 to 0.64)
South Africa	356 (279 to 467)	1.11 (0.92 to 1.39)	624 (508 to 800)	1.19 (0.99 to 1.49)	0.33% (0.25 to 0.41)
South Sudan	36 (27 to 49)	0.76 (0.63 to 0.95)	63 (48 to 84)	0.85 (0.7 to 1.05)	0.51% (0.43 to 0.6)
Spain	2329 (2162 to 2526)	4.26 (3.94 to 4.67)	5362 (4946 to 5787)	5.42 (4.97 to 5.94)	0.72% (0.41 to 1.03)
Sri Lanka	79 (63 to 105)	0.56 (0.46 to 0.71)	221 (183 to 267)	0.92 (0.78 to 1.12)	1.76% (1.68 to 1.84)
Sudan	80 (62 to 105)	0.55 (0.44 to 0.69)	250 (197 to 327)	0.9 (0.73 to 1.1)	1.61% (1.46 to 1.76)
Suriname	3 (2 to 4)	0.8 (0.66 to 1.01)	6 (5 to 8)	1.06 (0.88 to 1.32)	1.03% (0.92 to 1.13)
Sweden	709 (651 to 776)	4.57 (4.15 to 5.08)	508 (455 to 571)	2.57 (2.25 to 2.95)	-2.42% (-2.55 to -2.29)
Switzerland	258 (227 to 292)	2.59 (2.26 to 3)	526 (469 to 592)	3.24 (2.85 to 3.72)	0.89% (0.8 to 0.97)
Syrian Arab Republic	63 (49 to 82)	0.72 (0.59 to 0.89)	151 (121 to 189)	1.13 (0.93 to 1.39)	1.69% (1.59 to 1.8)
Taiwan (Province of China)	258 (214 to 319)	1.47 (1.24 to 1.78)	1234 (1117 to 1373)	3.46 (3.14 to 3.81)	3.38% (2.77 to 3.99)
Tajikistan	61 (45 to 84)	1.14 (0.92 to 1.49)	114 (88 to 151)	1.4 (1.16 to 1.76)	0.96% (0.73 to 1.2)
Thailand	318 (261 to 409)	0.74 (0.63 to 0.9)	1057 (903 to 1245)	1.17 (1.01 to 1.39)	1.64% (1.58 to 1.7)
Timor-Leste	2 (2 to 3)	0.4 (0.33 to 0.5)	7 (5 to 8)	0.66 (0.56 to 0.79)	2.1% (1.97 to 2.24)
Togo	26 (18 to 36)	0.75 (0.6 to 0.96)	63 (48 to 83)	0.96 (0.79 to 1.18)	0.81% (0.67 to 0.95)
Tokelau	0 (0 to 0)	0.48 (0.39 to 0.61)	0 (0 to 0)	0.74 (0.62 to 0.92)	1.6% (1.55 to 1.65)
Tonga	1 (0 to 1)	0.76 (0.65 to 0.91)	1 (1 to 1)	0.99 (0.86 to 1.18)	0.82% (0.75 to 0.9)
Trinidad and Tobago	12 (10 to 15)	1.23 (1.05 to 1.46)	22 (19 to 27)	1.37 (1.15 to 1.69)	0.32% (0.26 to 0.39)
Tunisia	56 (46 to 71)	0.88 (0.74 to 1.08)	184 (152 to 223)	1.5 (1.26 to 1.8)	1.84% (1.79 to 1.89)
Turkey	335 (281 to 411)	0.79 (0.68 to 0.94)	1357 (1172 to 1574)	1.59 (1.39 to 1.82)	2.61% (2.42 to 2.8)
Turkmenistan	46 (34 to 62)	1.3 (1.05 to 1.67)	87 (70 to 111)	1.86 (1.54 to 2.29)	1.5% (1.3 to 1.71)
Tuvalu	0 (0 to 0)	0.46 (0.37 to 0.57)	0 (0 to 0)	0.67 (0.56 to 0.83)	1.22% (1.14 to 1.29)
Uganda	78 (59 to 106)	0.66 (0.57 to 0.8)	253 (196 to 334)	0.98 (0.84 to 1.16)	1.52% (1.41 to 1.64)
Ukraine	3228 (2940 to 3567)	4.77 (4.31 to 5.31)	2923 (2696 to 3180)	4.21 (3.82 to 4.66)	-0.34% (-0.44 to -0.24)
United Arab Emirates	15 (12 to 22)	1.26 (1.07 to 1.54)	125 (90 to 177)	1.68 (1.42 to 2.01)	0.81% (0.68 to 0.94)
United Kingdom	3614 (3309 to 3973)	4.11 (3.7 to 4.61)	5303 (4878 to 5766)	4.44 (4.04 to 4.92)	0.33% (0.24 to 0.42)
United Republic of Tanzania	129 (98 to 174)	0.65 (0.54 to 0.81)	411 (322 to 535)	1.02 (0.86 to 1.22)	1.62% (1.51 to 1.73)
United States of America	21442 (19788 to 23418)	6.83 (6.26 to 7.54)	27053 (24999 to 29363)	5.34 (4.88 to 5.85)	-0.89% (-0.91 to -0.87)
United States Virgin Islands	1 (1 to 2)	1.34 (1.15 to 1.59)	3 (2 to 3)	1.74 (1.49 to 2.05)	0.94% (0.84 to 1.05)
Uruguay	152 (140 to 165)	3.87 (3.57 to 4.22)	285 (264 to 307)	5.2 (4.82 to 5.64)	1.03% (0.89 to 1.18)
Uzbekistan	242 (182 to 326)	1.21 (0.98 to 1.56)	489 (391 to 645)	1.66 (1.37 to 2.08)	1.23% (1.06 to 1.4)

Measure	1990		2019		1990-2019
	Cases (95% UI)	ASR (95% UI)	Cases (95% UI)	ASR (95% UI)	EAPC (95% UI)
Vanuatu	1 (0 to 1)	0.5 (0.41 to 0.62)	2 (1 to 2)	0.69 (0.58 to 0.82)	1.15% (1.1 to 1.2)
Venezuela (Bolivarian Republic of)	198 (168 to 243)	1.6 (1.42 to 1.83)	724 (645 to 821)	2.58 (2.31 to 2.91)	1.74% (1.51 to 1.96)
Viet Nam	275 (227 to 351)	0.56 (0.48 to 0.68)	811 (675 to 998)	0.91 (0.78 to 1.08)	1.69% (1.64 to 1.73)
Yemen	56 (44 to 76)	0.63 (0.52 to 0.78)	179 (143 to 235)	0.87 (0.73 to 1.07)	1.4% (1.32 to 1.47)
Zambia	42 (31 to 57)	0.73 (0.62 to 0.89)	123 (95 to 163)	1 (0.86 to 1.2)	1.21% (1.03 to 1.4)
Zimbabwe	62 (46 to 87)	0.73 (0.59 to 0.94)	93 (71 to 125)	0.74 (0.6 to 0.95)	-0.29% (-0.63 to 0.04)

UI=uncertainty interval; ASR= age-standardized rates; EAPC= estimated annual percentage changes.

Table S4 Deaths and age-standardized mortality rate of vascular intestinal disorders in 1990 and 2019, and estimated annual percentage change in age-standardized mortality rate from 1990 to 2019 in 204 countries and regions

Measure	1990		2019		1990-2019
	Deaths (95% UI)	ASR (95% UI)	Deaths (95% UI)	ASR (95% UI)	EAPC (95% UI)
Afghanistan	56 (32 to 80)	0.96 (0.58 to 1.36)	80 (49 to 111)	0.85 (0.56 to 1.18)	-0.35% (-0.4 to -0.3)
Albania	11 (10 to 15)	0.64 (0.54 to 0.88)	25 (18 to 33)	0.58 (0.42 to 0.79)	-0.17% (-0.29 to -0.04)
Algeria	78 (44 to 107)	0.93 (0.53 to 1.3)	190 (142 to 253)	0.74 (0.55 to 0.98)	-0.71% (-0.79 to -0.62)
American Samoa	0 (0 to 0)	0.68 (0.55 to 0.85)	0 (0 to 0)	0.67 (0.53 to 0.8)	0.06% (-0.03 to 0.14)
Andorra	1 (1 to 1)	1.77 (1.36 to 2.41)	3 (2 to 4)	1.94 (1.27 to 2.76)	0.3% (0.23 to 0.37)
Angola	44 (28 to 61)	1.45 (1.02 to 1.9)	136 (98 to 179)	1.7 (1.24 to 2.22)	0.67% (0.48 to 0.86)
Antigua and Barbuda	0 (0 to 0)	0.55 (0.48 to 0.63)	0 (0 to 1)	0.52 (0.43 to 0.62)	-0.39% (-0.67 to -0.11)
Argentina	733 (657 to 819)	2.43 (2.16 to 2.73)	1050 (923 to 1184)	1.89 (1.67 to 2.14)	-0.9% (-1.08 to -0.72)
Armenia	70 (63 to 79)	3.06 (2.74 to 3.46)	145 (119 to 173)	3.65 (3 to 4.34)	0.88% (0.61 to 1.16)
Australia	343 (303 to 372)	1.83 (1.61 to 1.99)	627 (518 to 721)	1.33 (1.1 to 1.51)	-1.21% (-1.28 to -1.14)
Austria	282 (255 to 305)	2.22 (2 to 2.38)	243 (203 to 275)	1.15 (0.97 to 1.29)	-2.71% (-3.17 to -2.26)
Azerbaijan	25 (20 to 31)	0.55 (0.43 to 0.67)	46 (33 to 67)	0.65 (0.43 to 1.09)	0.69% (0.58 to 0.8)
Bahamas	2 (2 to 3)	1.71 (1.46 to 1.95)	5 (4 to 6)	1.39 (1.11 to 1.71)	-0.65% (-0.82 to -0.49)
Bahrain	4 (4 to 5)	5.25 (4.22 to 6.24)	9 (7 to 11)	2.27 (1.77 to 2.82)	-3.94% (-4.69 to -3.18)
Bangladesh	585 (441 to 745)	1.46 (1.08 to 1.86)	1570 (1053 to 2052)	1.36 (0.94 to 1.76)	0% (-0.28 to 0.28)
Barbados	2 (2 to 3)	0.76 (0.63 to 0.88)	3 (3 to 4)	0.66 (0.52 to 0.82)	-0.65% (-0.8 to -0.5)
Belarus	267 (224 to 326)	2.14 (1.8 to 2.6)	517 (413 to 638)	3.14 (2.52 to 3.87)	1.54% (1.38 to 1.71)
Belgium	496 (445 to 540)	3.14 (2.82 to 3.41)	821 (693 to 939)	2.96 (2.54 to 3.35)	-0.78% (-1.17 to -0.39)
Belize	0 (0 to 1)	0.48 (0.4 to 0.59)	1 (1 to 2)	0.54 (0.45 to 0.64)	0.25% (-0.04 to 0.53)
Benin	18 (12 to 27)	0.71 (0.51 to 0.99)	41 (30 to 55)	0.87 (0.69 to 1.09)	0.65% (0.53 to 0.77)
Bermuda	1 (1 to 1)	1.74 (1.46 to 2.09)	1 (1 to 2)	0.83 (0.64 to 1.08)	-2.86% (-3.07 to -2.65)
Bhutan	2 (1 to 3)	1.16 (0.83 to 1.67)	8 (5 to 15)	1.74 (1.15 to 3.2)	1.47% (1.32 to 1.62)
Bolivia (Plurinational State of)	45 (29 to 64)	1.56 (1.1 to 2.1)	119 (88 to 154)	1.65 (1.24 to 2.1)	0.06% (-0.04 to 0.15)
Bosnia and Herzegovina	36 (31 to 41)	1.15 (0.99 to 1.3)	77 (59 to 99)	1.32 (1.02 to 1.67)	0.38% (0.16 to 0.6)
Botswana	5 (3 to 7)	0.99 (0.67 to 1.42)	13 (9 to 18)	1.13 (0.82 to 1.55)	-0.09% (-0.5 to 0.31)
Brazil	2249 (2113 to 2363)	2.94 (2.72 to 3.11)	4399 (3974 to 4858)	1.95 (1.75 to 2.16)	-1.54% (-1.61 to -1.47)
Brunei Darussalam	1 (1 to 1)	1.49 (1.09 to 2.03)	3 (2 to 3)	1.58 (1.33 to 1.84)	0.32% (0.24 to 0.4)
Bulgaria	147 (130 to 163)	1.31 (1.15 to 1.45)	281 (223 to 344)	1.84 (1.47 to 2.26)	2.38% (1.86 to 2.9)
Burkina Faso	39 (27 to 57)	0.85 (0.67 to 1.06)	97 (57 to 158)	1.12 (0.68 to 1.75)	1.3% (1.07 to 1.53)
Burundi	28 (17 to 41)	1.38 (0.81 to 2.09)	55 (37 to 80)	1.57 (1.05 to 2.26)	0.25% (0.03 to 0.46)
Cabo Verde	1 (1 to 2)	0.53 (0.4 to 0.7)	3 (3 to 4)	0.81 (0.65 to 1.02)	0.51% (0.06 to 0.97)
Cambodia	22 (17 to 29)	0.66 (0.5 to 0.84)	70 (54 to 86)	0.78 (0.61 to 0.98)	0.55% (0.47 to 0.62)
Cameroon	34 (25 to 46)	0.8 (0.62 to 1.01)	103 (61 to 163)	0.98 (0.6 to 1.53)	0.7% (0.54 to 0.87)
Canada	699 (628 to 771)	2.2 (1.97 to 2.45)	1410 (1179 to 1702)	1.87 (1.57 to 2.23)	-0.63% (-0.72 to -0.54)
Central African Republic	19 (11 to 31)	2.02 (1.27 to 3.05)	33 (19 to 53)	1.97 (1.3 to 2.86)	-0.05% (-0.15 to 0.05)
Chad	30 (18 to 47)	0.92 (0.63 to 1.26)	63 (40 to 91)	1.09 (0.77 to 1.47)	0.57% (0.43 to 0.71)
Chile	259 (237 to 285)	2.99 (2.7 to 3.3)	538 (463 to 611)	2.25 (1.94 to 2.56)	-1.03% (-1.14 to -0.91)
China	2925 (2494 to 3686)	0.46 (0.4 to 0.57)	6413 (5193 to 7406)	0.37 (0.3 to 0.43)	-0.68% (-0.74 to -0.62)

Measure	1990		2019		1990-2019
	Deaths (95% UI)	ASR (95% UI)	Deaths (95% UI)	ASR (95% UI)	EAPC (95% UI)
Colombia	330 (303 to 355)	2.14 (1.94 to 2.31)	1080 (827 to 1366)	1.99 (1.53 to 2.53)	-0.3% (-0.56 to -0.03)
Comoros	3 (2 to 5)	1.75 (1.19 to 2.44)	7 (5 to 10)	1.74 (1.22 to 2.41)	-0.14% (-0.24 to -0.04)
Congo	18 (12 to 25)	2.16 (1.5 to 2.86)	37 (27 to 50)	1.98 (1.48 to 2.53)	-0.27% (-0.39 to -0.15)
Cook Islands	0 (0 to 0)	1.15 (0.93 to 1.49)	0 (0 to 0)	1.06 (0.87 to 1.31)	-0.37% (-0.48 to -0.27)
Costa Rica	56 (49 to 64)	3.53 (3.03 to 4.05)	205 (151 to 263)	4.02 (2.94 to 5.17)	0.02% (-0.23 to 0.28)
Côte d'Ivoire	35 (20 to 57)	0.8 (0.54 to 1.09)	81 (54 to 114)	0.88 (0.62 to 1.22)	0.1% (-0.14 to 0.35)
Croatia	182 (164 to 201)	3.1 (2.8 to 3.44)	227 (176 to 285)	2.33 (1.8 to 2.91)	-1.14% (-1.43 to -0.85)
Cuba	215 (184 to 250)	2.14 (1.84 to 2.5)	391 (309 to 498)	1.98 (1.56 to 2.52)	-0.29% (-0.47 to -0.12)
Cyprus	12 (8 to 20)	2.04 (1.27 to 3.7)	24 (19 to 32)	1.44 (1.16 to 2.05)	-1.54% (-1.66 to -1.42)
Czechia	266 (244 to 296)	1.94 (1.78 to 2.17)	277 (220 to 349)	1.24 (0.99 to 1.56)	-1.46% (-1.62 to -1.29)
Democratic People's Republic of Korea	54 (40 to 70)	0.45 (0.34 to 0.58)	124 (81 to 162)	0.43 (0.28 to 0.56)	-0.08% (-0.16 to 0)
Democratic Republic of the Congo	343 (137 to 625)	2.85 (1.25 to 4.97)	583 (299 to 980)	2.06 (1.06 to 3.35)	-1.3% (-1.52 to -1.09)
Denmark	166 (147 to 184)	1.93 (1.73 to 2.14)	162 (138 to 188)	1.28 (1.09 to 1.47)	-2.46% (-2.98 to -1.94)
Djibouti	1 (1 to 2)	1.42 (1.03 to 2.02)	6 (4 to 9)	1.5 (1.02 to 2.14)	0.08% (0.04 to 0.12)
Dominica	1 (1 to 2)	1.92 (1.66 to 2.22)	1 (1 to 2)	1.64 (1.33 to 2.03)	-0.45% (-0.51 to -0.38)
Dominican Republic	25 (21 to 31)	0.73 (0.62 to 0.97)	71 (53 to 96)	0.82 (0.62 to 1.1)	0.91% (0.57 to 1.26)
Ecuador	69 (60 to 79)	1.44 (1.26 to 1.65)	253 (201 to 330)	1.92 (1.53 to 2.5)	1.7% (1.29 to 2.12)
Egypt	140 (111 to 210)	0.64 (0.51 to 1.04)	299 (151 to 485)	0.65 (0.33 to 1.1)	0.27% (0.15 to 0.39)
El Salvador	23 (20 to 26)	0.83 (0.72 to 0.93)	64 (48 to 83)	1.01 (0.75 to 1.31)	0.94% (0.76 to 1.11)
Equatorial Guinea	3 (1 to 5)	1.81 (0.97 to 2.97)	9 (5 to 14)	2.36 (1.36 to 3.75)	1.05% (0.89 to 1.21)
Eritrea	9 (6 to 13)	1.23 (0.85 to 1.77)	34 (24 to 46)	1.83 (1.32 to 2.48)	1.22% (0.98 to 1.45)
Estonia	91 (81 to 104)	4.52 (4.03 to 5.17)	120 (96 to 150)	3.85 (3.08 to 4.8)	-0.9% (-1.33 to -0.47)
Eswatini	2 (1 to 3)	0.66 (0.48 to 0.96)	5 (3 to 7)	0.99 (0.7 to 1.34)	1.64% (1.4 to 1.88)
Ethiopia	288 (175 to 468)	1.84 (1.28 to 2.67)	608 (424 to 857)	1.82 (1.25 to 2.56)	-0.19% (-0.42 to 0.04)
Fiji	2 (1 to 2)	0.64 (0.49 to 0.83)	4 (3 to 5)	0.7 (0.53 to 0.89)	0.26% (0.19 to 0.33)
Finland	176 (152 to 205)	2.44 (2.08 to 2.82)	244 (207 to 279)	1.66 (1.41 to 1.88)	-1.65% (-1.82 to -1.47)
France	2632 (2356 to 2922)	2.91 (2.6 to 3.22)	3231 (2658 to 3754)	1.85 (1.57 to 2.12)	-1.81% (-1.9 to -1.71)
Gabon	9 (6 to 14)	1.96 (1.24 to 2.75)	16 (11 to 22)	1.97 (1.37 to 2.66)	-0.06% (-0.23 to 0.11)
Gambia	4 (2 to 5)	0.97 (0.69 to 1.33)	8 (6 to 11)	0.93 (0.67 to 1.27)	-0.53% (-0.77 to -0.28)
Georgia	26 (22 to 31)	0.5 (0.42 to 0.6)	62 (47 to 77)	0.96 (0.74 to 1.2)	3.32% (2.45 to 4.19)
Germany	4211 (3746 to 4679)	3.13 (2.79 to 3.48)	4757 (4123 to 5422)	2.12 (1.85 to 2.39)	-1.76% (-1.99 to -1.52)
Ghana	62 (44 to 86)	0.94 (0.69 to 1.35)	131 (99 to 171)	0.94 (0.72 to 1.22)	-0.33% (-0.66 to 0.01)
Greece	152 (136 to 168)	1.03 (0.92 to 1.14)	346 (285 to 412)	1.14 (0.96 to 1.34)	0.07% (-0.24 to 0.38)
Greenland	1 (1 to 1)	4.02 (3.23 to 5.02)	2 (2 to 3)	3.83 (2.96 to 4.72)	-0.47% (-0.7 to -0.25)
Grenada	1 (0 to 1)	0.64 (0.54 to 0.76)	1 (1 to 1)	0.72 (0.61 to 0.85)	0.43% (0.21 to 0.64)
Guam	0 (0 to 0)	0.8 (0.65 to 0.99)	1 (1 to 1)	0.6 (0.46 to 0.73)	-1% (-1.4 to -0.59)
Guatemala	28 (23 to 34)	0.91 (0.79 to 1.06)	75 (58 to 93)	0.79 (0.62 to 0.98)	-0.73% (-0.84 to -0.61)
Guinea	29 (16 to 48)	0.75 (0.47 to 1.07)	46 (28 to 70)	0.83 (0.53 to 1.2)	0.34% (0.2 to 0.47)
Guinea-Bissau	5 (2 to 8)	1.05 (0.67 to 1.56)	7 (5 to 11)	1.17 (0.84 to 1.57)	0.41% (0.29 to 0.54)
Guyana	4 (3 to 4)	1.12 (0.94 to 1.3)	6 (4 to 7)	1.04 (0.8 to 1.31)	-0.01% (-0.26 to 0.25)

Measure	1990		2019		1990-2019
	Deaths (95% UI)	ASR (95% UI)	Deaths (95% UI)	ASR (95% UI)	EAPC (95% UI)
Haiti	39 (27 to 51)	1.51 (1.1 to 2.13)	62 (42 to 88)	1.07 (0.73 to 1.47)	-1.11% (-1.27 to -0.95)
Honduras	63 (48 to 78)	3.34 (2.4 to 4.37)	237 (174 to 300)	4.8 (3.59 to 6.1)	1.55% (1.32 to 1.79)
Hungary	472 (442 to 501)	3.35 (3.14 to 3.56)	575 (475 to 691)	2.81 (2.31 to 3.37)	-0.93% (-1.12 to -0.74)
Iceland	5 (5 to 6)	1.79 (1.54 to 2.01)	7 (6 to 8)	1.1 (0.89 to 1.29)	-2.2% (-2.46 to -1.93)
India	3531 (2731 to 4685)	1.15 (0.9 to 1.48)	11373 (7930 to 16058)	1.22 (0.87 to 1.7)	-0.04% (-0.21 to 0.12)
Indonesia	719 (583 to 873)	1.08 (0.83 to 1.34)	1878 (1538 to 2215)	1.35 (1.09 to 1.59)	0.74% (0.67 to 0.81)
Iran (Islamic Republic of)	109 (79 to 147)	0.63 (0.42 to 0.88)	323 (268 to 366)	0.52 (0.43 to 0.59)	-0.36% (-0.68 to -0.03)
Iraq	31 (24 to 41)	0.45 (0.34 to 0.58)	75 (57 to 93)	0.4 (0.3 to 0.5)	-0.27% (-0.44 to -0.11)
Ireland	74 (68 to 81)	1.82 (1.67 to 1.98)	137 (115 to 156)	1.75 (1.48 to 1.99)	0.33% (0.11 to 0.55)
Israel	97 (87 to 108)	2.13 (1.88 to 2.37)	242 (202 to 294)	1.9 (1.59 to 2.28)	-0.77% (-0.91 to -0.63)
Italy	1637 (1504 to 1720)	1.83 (1.67 to 1.93)	2353 (1988 to 2585)	1.28 (1.11 to 1.4)	-1.37% (-1.47 to -1.26)
Jamaica	12 (11 to 15)	0.68 (0.58 to 0.79)	19 (14 to 23)	0.6 (0.46 to 0.74)	-0.53% (-0.91 to -0.15)
Japan	1134 (1050 to 1186)	0.72 (0.66 to 0.75)	3710 (2901 to 4373)	0.75 (0.61 to 0.85)	0.34% (0.19 to 0.49)
Jordan	17 (13 to 21)	1.68 (1.31 to 2.13)	46 (37 to 57)	0.96 (0.76 to 1.19)	-2.5% (-2.84 to -2.16)
Kazakhstan	197 (172 to 236)	1.78 (1.55 to 2.15)	225 (190 to 267)	1.5 (1.27 to 1.78)	-0.93% (-1.3 to -0.55)
Kenya	121 (68 to 191)	1.85 (1.01 to 2.98)	381 (215 to 586)	2.4 (1.34 to 3.75)	1.05% (0.97 to 1.13)
Kiribati	1 (1 to 1)	2.29 (1.7 to 3.04)	1 (1 to 2)	2.12 (1.54 to 2.83)	-0.31% (-0.4 to -0.23)
Kuwait	5 (4 to 6)	1.16 (0.97 to 1.37)	19 (15 to 23)	0.98 (0.77 to 1.19)	-0.18% (-0.48 to 0.12)
Kyrgyzstan	17 (16 to 20)	0.59 (0.53 to 0.67)	20 (17 to 24)	0.53 (0.45 to 0.62)	-0.58% (-0.81 to -0.35)
Lao People's Democratic Republic	9 (6 to 13)	0.58 (0.41 to 0.78)	22 (15 to 29)	0.66 (0.47 to 0.89)	0.35% (0.23 to 0.46)
Latvia	166 (151 to 185)	4.67 (4.24 to 5.19)	206 (170 to 248)	4.42 (3.68 to 5.28)	-0.52% (-0.85 to -0.18)
Lebanon	13 (9 to 17)	0.72 (0.5 to 0.93)	33 (20 to 48)	0.65 (0.39 to 0.94)	-0.32% (-0.4 to -0.24)
Lesotho	6 (4 to 8)	0.66 (0.46 to 0.95)	10 (7 to 14)	0.92 (0.66 to 1.25)	1.45% (1.26 to 1.64)
Liberia	10 (6 to 16)	0.82 (0.54 to 1.1)	16 (11 to 23)	0.88 (0.63 to 1.17)	0.46% (0.33 to 0.59)
Libya	12 (7 to 20)	0.79 (0.41 to 1.27)	27 (17 to 41)	0.64 (0.4 to 0.96)	-0.61% (-0.68 to -0.54)
Lithuania	119 (107 to 133)	2.64 (2.37 to 2.97)	303 (241 to 377)	4.46 (3.57 to 5.51)	2.37% (2.01 to 2.73)
Luxembourg	18 (16 to 20)	3.37 (2.99 to 3.75)	26 (21 to 31)	2.31 (1.89 to 2.76)	-1.65% (-1.9 to -1.39)
Madagascar	46 (29 to 66)	1.08 (0.68 to 1.61)	104 (75 to 144)	1.34 (0.96 to 1.87)	0.8% (0.71 to 0.89)
Malawi	42 (31 to 52)	1.38 (0.92 to 1.91)	93 (63 to 128)	1.6 (1.06 to 2.21)	0.54% (0.45 to 0.63)
Malaysia	69 (59 to 82)	0.92 (0.77 to 1.09)	342 (256 to 463)	1.65 (1.25 to 2.24)	1.56% (1.13 to 2)
Maldives	0 (0 to 1)	0.67 (0.39 to 1.01)	1 (1 to 2)	0.62 (0.49 to 0.79)	-0.72% (-0.86 to -0.58)
Mali	35 (17 to 61)	0.72 (0.46 to 1.02)	80 (48 to 124)	0.91 (0.63 to 1.33)	0.93% (0.84 to 1.02)
Malta	7 (6 to 7)	1.63 (1.42 to 1.82)	13 (11 to 15)	1.26 (1.04 to 1.48)	-1.29% (-1.6 to -0.98)
Marshall Islands	0 (0 to 0)	0.95 (0.69 to 1.22)	0 (0 to 0)	0.91 (0.58 to 1.4)	-0.24% (-0.38 to -0.1)
Mauritania	10 (7 to 13)	0.98 (0.73 to 1.29)	15 (10 to 21)	0.81 (0.57 to 1.09)	-0.89% (-1.04 to -0.74)
Mauritius	3 (2 to 3)	0.43 (0.38 to 0.49)	7 (6 to 9)	0.48 (0.38 to 0.59)	0.28% (0.13 to 0.44)
Mexico	972 (911 to 1014)	2.66 (2.46 to 2.79)	2603 (2211 to 3028)	2.44 (2.07 to 2.84)	-0.46% (-0.54 to -0.37)
Micronesia (Federated States of)	0 (0 to 0)	0.92 (0.68 to 1.25)	1 (0 to 1)	1.01 (0.61 to 1.63)	0.29% (0.21 to 0.37)
Monaco	1 (1 to 2)	1.47 (1.13 to 1.84)	2 (1 to 2)	1.5 (1.16 to 1.8)	0.09% (0.01 to 0.18)
Mongolia	7 (5 to 8)	0.72 (0.58 to 0.88)	11 (8 to 14)	0.63 (0.49 to 0.88)	-0.65% (-0.77 to -0.53)

Measure	1990		2019		1990-2019
	Deaths (95% UI)	ASR (95% UI)	Deaths (95% UI)	ASR (95% UI)	EAPC (95% UI)
Montenegro	23 (18 to 29)	3.89 (3.06 to 4.89)	42 (35 to 53)	4.42 (3.67 to 5.48)	0.66% (0.52 to 0.79)
Morocco	78 (49 to 113)	0.74 (0.44 to 1.13)	181 (133 to 230)	0.75 (0.53 to 0.99)	-0.01% (-0.19 to 0.17)
Mozambique	72 (54 to 91)	1.49 (1.16 to 1.9)	203 (145 to 278)	2.25 (1.64 to 3.07)	1.65% (1.53 to 1.77)
Myanmar	60 (34 to 99)	0.3 (0.18 to 0.45)	127 (91 to 179)	0.32 (0.23 to 0.43)	0.34% (0.2 to 0.48)
Namibia	6 (5 to 8)	0.99 (0.72 to 1.31)	15 (11 to 19)	1.14 (0.85 to 1.48)	0.43% (0.28 to 0.58)
Nauru	0 (0 to 0)	1 (0.68 to 1.38)	0 (0 to 0)	1.03 (0.69 to 1.48)	0.07% (-0.12 to 0.25)
Nepal	123 (87 to 174)	1.77 (1.2 to 2.66)	343 (257 to 479)	2 (1.5 to 2.77)	0.16% (0.03 to 0.28)
Netherlands	432 (388 to 468)	2.1 (1.9 to 2.28)	620 (530 to 723)	1.67 (1.44 to 1.94)	-1.12% (-1.41 to -0.84)
New Zealand	75 (68 to 83)	1.94 (1.74 to 2.14)	112 (93 to 133)	1.3 (1.09 to 1.53)	-1.49% (-1.79 to -1.19)
Nicaragua	10 (9 to 11)	0.74 (0.62 to 0.84)	38 (31 to 47)	1.06 (0.88 to 1.27)	1.19% (0.78 to 1.6)
Niger	33 (14 to 65)	0.87 (0.55 to 1.25)	78 (44 to 124)	1 (0.65 to 1.43)	0.55% (0.47 to 0.62)
Nigeria	412 (286 to 605)	0.91 (0.59 to 1.45)	870 (503 to 1519)	1.12 (0.65 to 2)	1.04% (0.83 to 1.26)
Niue	0 (0 to 0)	0.79 (0.61 to 1.02)	0 (0 to 0)	0.86 (0.61 to 1.14)	0.32% (0.21 to 0.43)
North Macedonia	30 (23 to 35)	1.8 (1.39 to 2.12)	40 (31 to 51)	1.38 (1.1 to 1.73)	-1.27% (-1.53 to -1.02)
Northern Mariana Islands	0 (0 to 0)	1.23 (1.01 to 1.49)	0 (0 to 0)	1 (0.82 to 1.19)	-0.84% (-0.98 to -0.7)
Norway	118 (106 to 133)	1.55 (1.4 to 1.74)	121 (104 to 144)	1.11 (0.96 to 1.3)	-1.59% (-1.77 to -1.41)
Oman	3 (2 to 4)	0.72 (0.49 to 1.02)	7 (5 to 8)	0.71 (0.57 to 0.84)	0.33% (0.13 to 0.53)
Pakistan	725 (446 to 1147)	1.5 (0.92 to 2.41)	1285 (841 to 1744)	1.65 (1.1 to 2.19)	0.42% (0.32 to 0.51)
Palau	0 (0 to 0)	0.68 (0.47 to 0.97)	0 (0 to 0)	0.79 (0.47 to 1.12)	0.57% (0.45 to 0.68)
Palestine	7 (5 to 9)	0.92 (0.67 to 1.21)	16 (13 to 20)	0.99 (0.8 to 1.21)	0.12% (-0.03 to 0.27)
Panama	21 (18 to 24)	1.47 (1.27 to 1.68)	58 (43 to 74)	1.37 (1.03 to 1.75)	-0.44% (-0.6 to -0.27)
Papua New Guinea	4 (3 to 6)	0.28 (0.2 to 0.37)	15 (10 to 20)	0.35 (0.25 to 0.47)	0.84% (0.74 to 0.94)
Paraguay	33 (26 to 38)	1.61 (1.26 to 1.9)	97 (71 to 133)	1.88 (1.38 to 2.57)	0.85% (0.71 to 0.99)
Peru	128 (101 to 159)	1.06 (0.86 to 1.25)	215 (159 to 289)	0.66 (0.49 to 0.89)	-1.85% (-2.12 to -1.59)
Philippines	94 (73 to 116)	0.43 (0.33 to 0.52)	239 (198 to 283)	0.4 (0.33 to 0.47)	-0.2% (-0.49 to 0.1)
Poland	421 (393 to 450)	1.02 (0.94 to 1.09)	1510 (1250 to 1759)	2.03 (1.69 to 2.37)	2.03% (0.94 to 3.13)
Portugal	271 (243 to 295)	2.1 (1.88 to 2.3)	856 (722 to 973)	2.9 (2.47 to 3.27)	1.16% (0.86 to 1.47)
Puerto Rico	40 (35 to 45)	1.13 (1 to 1.29)	52 (39 to 67)	0.65 (0.48 to 0.83)	-2.67% (-2.92 to -2.42)
Qatar	1 (0 to 1)	1.05 (0.78 to 1.41)	3 (2 to 4)	1.03 (0.75 to 1.39)	0.26% (0.02 to 0.49)
Republic of Korea	135 (104 to 184)	0.61 (0.47 to 0.82)	632 (480 to 754)	0.76 (0.58 to 0.91)	1.53% (1.25 to 1.82)
Republic of Moldova	76 (68 to 84)	1.83 (1.65 to 2.02)	184 (153 to 216)	3.17 (2.64 to 3.72)	2.67% (2.24 to 3.11)
Romania	478 (428 to 530)	1.82 (1.62 to 2.02)	1006 (823 to 1212)	2.57 (2.11 to 3.1)	1.54% (1.32 to 1.75)
Russian Federation	5074 (4394 to 5862)	3.04 (2.62 to 3.49)	11232 (9790 to 12624)	4.74 (4.13 to 5.33)	1.87% (1.62 to 2.13)
Rwanda	41 (25 to 59)	1.81 (1.05 to 2.75)	72 (38 to 108)	1.6 (0.85 to 2.38)	-0.91% (-1.21 to -0.62)
Saint Kitts and Nevis	1 (1 to 1)	2.4 (2.01 to 2.94)	1 (1 to 1)	1.71 (1.37 to 2.18)	-1.06% (-1.24 to -0.89)
Saint Lucia	1 (1 to 1)	1.41 (1.21 to 1.62)	2 (2 to 3)	1.11 (0.9 to 1.37)	-1.28% (-1.64 to -0.91)
Saint Vincent and the Grenadines	0 (0 to 0)	0.52 (0.45 to 0.61)	1 (0 to 1)	0.42 (0.34 to 0.5)	-0.57% (-1 to -0.14)
Samoa	1 (0 to 1)	0.71 (0.55 to 0.94)	1 (1 to 1)	0.73 (0.58 to 0.92)	0.18% (0.09 to 0.26)
San Marino	0 (0 to 0)	0.54 (0.42 to 0.65)	0 (0 to 1)	0.54 (0.36 to 0.78)	0.37% (0.24 to 0.5)
Sao Tome and Principe	1 (0 to 1)	0.78 (0.54 to 1.09)	1 (0 to 1)	0.74 (0.53 to 1.19)	-0.15% (-0.48 to 0.19)
Saudi Arabia	45 (30 to 65)	1.02 (0.7 to 1.49)	80 (54 to 132)	0.72 (0.5 to 1.35)	-1.44% (-1.58 to -1.3)

Measure	1990		2019		1990-2019
	Deaths (95% UI)	ASR (95% UI)	Deaths (95% UI)	ASR (95% UI)	EAPC (95% UI)
Senegal	36 (18 to 60)	0.98 (0.57 to 1.45)	72 (44 to 110)	1.06 (0.66 to 1.63)	0.21% (0.09 to 0.33)
Serbia	301 (247 to 367)	2.99 (2.45 to 3.61)	464 (368 to 572)	2.89 (2.3 to 3.52)	-0.02% (-0.33 to 0.29)
Seychelles	0 (0 to 0)	0.63 (0.47 to 0.81)	1 (1 to 1)	0.78 (0.54 to 1.14)	0.34% (0.06 to 0.62)
Sierra Leone	15 (9 to 25)	0.67 (0.45 to 0.94)	30 (19 to 45)	0.84 (0.57 to 1.23)	1.05% (0.84 to 1.25)
Singapore	12 (10 to 13)	0.63 (0.56 to 0.69)	58 (48 to 67)	0.82 (0.68 to 0.94)	1.71% (1.23 to 2.18)
Slovakia	45 (39 to 51)	0.78 (0.68 to 0.89)	126 (98 to 162)	1.38 (1.07 to 1.77)	2.9% (2.56 to 3.24)
Slovenia	86 (66 to 109)	3.59 (2.78 to 4.56)	139 (105 to 194)	2.69 (2.04 to 3.68)	-1.1% (-1.36 to -0.85)
Solomon Islands	1 (0 to 1)	0.44 (0.32 to 0.62)	1 (1 to 2)	0.51 (0.39 to 0.65)	0.49% (0.34 to 0.64)
Somalia	21 (11 to 33)	1.13 (0.65 to 1.59)	53 (32 to 78)	1.12 (0.68 to 1.57)	0.03% (-0.04 to 0.09)
South Africa	141 (112 to 182)	0.7 (0.56 to 0.93)	324 (272 to 375)	0.82 (0.69 to 0.95)	0.44% (0.25 to 0.62)
South Sudan	31 (20 to 49)	1.5 (1.02 to 2.32)	47 (31 to 80)	1.59 (1.06 to 2.58)	0.23% (0.2 to 0.27)
Spain	1688 (1532 to 1817)	3.13 (2.81 to 3.38)	3650 (3047 to 4370)	2.9 (2.47 to 3.42)	-0.61% (-1.01 to -0.2)
Sri Lanka	38 (29 to 50)	0.44 (0.33 to 0.58)	94 (69 to 123)	0.44 (0.33 to 0.57)	0.25% (0.01 to 0.49)
Sudan	43 (21 to 67)	0.56 (0.28 to 0.92)	94 (51 to 157)	0.63 (0.33 to 1.06)	0.4% (0.36 to 0.45)
Suriname	1 (1 to 2)	0.61 (0.54 to 0.69)	3 (3 to 4)	0.6 (0.48 to 0.72)	-0.19% (-0.37 to -0.01)
Sweden	332 (296 to 362)	1.95 (1.74 to 2.13)	268 (229 to 303)	1.05 (0.91 to 1.19)	-2.3% (-2.41 to -2.2)
Switzerland	120 (102 to 147)	1.05 (0.89 to 1.28)	251 (204 to 321)	1.19 (0.98 to 1.49)	0.2% (-0.2 to 0.59)
Syrian Arab Republic	22 (15 to 30)	0.53 (0.34 to 0.74)	42 (29 to 56)	0.47 (0.33 to 0.61)	-0.53% (-0.67 to -0.39)
Taiwan (Province of China)	57 (52 to 64)	0.47 (0.42 to 0.53)	576 (449 to 726)	1.44 (1.12 to 1.81)	4.31% (3.31 to 5.33)
Tajikistan	15 (11 to 18)	0.51 (0.36 to 0.63)	29 (22 to 39)	0.88 (0.66 to 1.17)	2.09% (1.93 to 2.24)
Thailand	209 (175 to 249)	0.78 (0.64 to 0.94)	731 (540 to 980)	0.76 (0.56 to 1.02)	-0.2% (-0.34 to -0.05)
Timor-Leste	1 (1 to 1)	0.46 (0.31 to 0.68)	4 (3 to 6)	0.62 (0.41 to 0.91)	1.18% (1.07 to 1.3)
Togo	11 (7 to 17)	0.81 (0.59 to 1.04)	31 (21 to 44)	1.02 (0.71 to 1.39)	0.9% (0.76 to 1.05)
Tokelau	0 (0 to 0)	0.66 (0.48 to 0.88)	0 (0 to 0)	0.76 (0.58 to 0.98)	0.65% (0.54 to 0.76)
Tonga	0 (0 to 1)	0.99 (0.7 to 1.33)	1 (1 to 1)	1.06 (0.82 to 1.37)	0.2% (0.06 to 0.34)
Trinidad and Tobago	10 (9 to 12)	1.39 (1.18 to 1.61)	15 (11 to 19)	0.83 (0.6 to 1.11)	-2.4% (-2.72 to -2.08)
Tunisia	23 (15 to 32)	0.59 (0.39 to 0.81)	64 (43 to 90)	0.58 (0.39 to 0.8)	0.04% (0 to 0.09)
Turkey	333 (259 to 413)	1.04 (0.82 to 1.27)	682 (544 to 835)	0.83 (0.66 to 1.02)	-0.59% (-0.81 to -0.36)
Turkmenistan	11 (10 to 12)	0.66 (0.57 to 0.76)	29 (23 to 37)	0.89 (0.71 to 1.13)	0.98% (0.77 to 1.2)
Tuvalu	0 (0 to 0)	0.77 (0.54 to 1.03)	0 (0 to 0)	0.8 (0.57 to 1.11)	0% (-0.11 to 0.12)
Uganda	79 (52 to 108)	1.51 (0.98 to 2.08)	202 (141 to 269)	1.81 (1.27 to 2.37)	0.49% (0.39 to 0.6)
Ukraine	2094 (1901 to 2308)	3.08 (2.81 to 3.4)	2630 (2206 to 3092)	3.37 (2.82 to 3.95)	0.02% (-0.11 to 0.16)
United Arab Emirates	3 (1 to 5)	1.01 (0.45 to 1.67)	19 (11 to 35)	0.8 (0.46 to 1.44)	-1.02% (-1.55 to -0.49)
United Kingdom	1825 (1566 to 1932)	1.95 (1.68 to 2.06)	3745 (3297 to 4200)	2.65 (2.36 to 2.95)	1.31% (1.18 to 1.44)
United Republic of Tanzania	110 (84 to 136)	1.25 (0.9 to 1.63)	323 (229 to 432)	1.57 (1.11 to 2.11)	0.9% (0.74 to 1.07)
United States of America	7806 (7073 to 8222)	2.32 (2.11 to 2.44)	11085 (9892 to 11827)	1.86 (1.68 to 1.98)	-1.1% (-1.33 to -0.87)
United States Virgin Islands	1 (1 to 1)	1.26 (1.01 to 1.52)	2 (2 to 2)	1.1 (0.89 to 1.3)	-0.35% (-0.58 to -0.12)
Uruguay	196 (175 to 215)	5.06 (4.52 to 5.57)	301 (256 to 343)	4.79 (4.17 to 5.44)	-0.23% (-0.35 to -0.12)
Uzbekistan	47 (31 to 60)	0.45 (0.28 to 0.59)	83 (68 to 99)	0.71 (0.59 to 0.85)	1.78% (1.62 to 1.93)

Measure	1990		2019		1990-2019
	Deaths (95% UI)	ASR (95% UI)	Deaths (95% UI)	ASR (95% UI)	EAPC (95% UI)
Vanuatu	0 (0 to 0)	0.6 (0.39 to 0.94)	1 (1 to 2)	0.76 (0.57 to 1.02)	0.92% (0.79 to 1.05)
Venezuela (Bolivarian Republic of)	193 (171 to 217)	2.21 (1.94 to 2.49)	673 (499 to 864)	2.5 (1.86 to 3.23)	0.47% (0.3 to 0.64)
Viet Nam	374 (273 to 516)	1.12 (0.82 to 1.56)	613 (452 to 826)	0.84 (0.62 to 1.14)	-1.45% (-1.64 to -1.25)
Yemen	28 (17 to 46)	0.78 (0.45 to 1.35)	74 (50 to 111)	0.72 (0.47 to 1.11)	-0.18% (-0.23 to -0.12)
Zambia	39 (30 to 50)	1.72 (1.34 to 2.2)	87 (65 to 112)	1.69 (1.26 to 2.17)	-0.28% (-0.43 to -0.13)
Zimbabwe	19 (13 to 27)	0.62 (0.43 to 0.89)	42 (22 to 70)	0.8 (0.43 to 1.32)	0.92% (0.85 to 0.98)

UI=uncertainty interval; ASR= age-standardized rates; EAPC= estimated annual percentage changes.

Table S5 DALYs and age-standardized DALYs rate of vascular intestinal disorders in 1990 and 2019, and estimated annual percentage change in age-standardized DALYs rate from 1990 to 2019 in 204 countries and regions

Measure	1990		2019		1990-2019
	Years (95% UI)	ASR (95% UI)	Years (95% UI)	ASR (95% UI)	EAPC (95% UI)
Afghanistan	1258 (636 to 1955)	18.19 (9.95 to 26.74)	1975 (1130 to 3081)	15.72 (9.63 to 22.07)	-0.42% (-0.46 to -0.38)
Albania	261 (220 to 370)	12.03 (10.33 to 16.08)	413 (291 to 564)	10.21 (7.3 to 13.89)	-0.35% (-0.53 to -0.16)
Algeria	1686 (978 to 2322)	15.2 (8.82 to 20.85)	3670 (2761 to 4810)	11.9 (8.97 to 15.65)	-0.8% (-0.89 to -0.72)
American Samoa	3 (2 to 4)	12.8 (10.5 to 15.9)	6 (5 to 7)	12.78 (10.33 to 15.35)	0.18% (0.06 to 0.3)
Andorra	15 (11 to 21)	29.45 (22 to 40.49)	44 (29 to 63)	31.42 (20.29 to 45.09)	0.2% (0.15 to 0.25)
Angola	1332 (722 to 2189)	29.12 (18.94 to 39.35)	3632 (2587 to 4883)	31.13 (22.38 to 40.72)	0.34% (0.15 to 0.53)
Antigua and Barbuda	6 (5 to 7)	10.77 (9.53 to 12.19)	9 (7 to 11)	9.3 (7.79 to 11.14)	-0.71% (-0.98 to -0.44)
Argentina	14195 (12844 to 15769)	44.44 (40.37 to 49.26)	18329 (16379 to 20569)	34.08 (30.46 to 38.22)	-0.92% (-1.06 to -0.77)
Armenia	1528 (1376 to 1706)	58.52 (52.76 to 65.38)	2440 (2010 to 2916)	60.15 (50.11 to 71.53)	0.26% (0 to 0.52)
Australia	5824 (5284 to 6268)	29.99 (27.25 to 32.26)	8738 (7545 to 9813)	20.3 (17.76 to 22.74)	-1.48% (-1.55 to -1.42)
Austria	4270 (3939 to 4557)	34.54 (32 to 36.75)	3355 (2897 to 3738)	17.93 (15.68 to 19.95)	-2.66% (-3.02 to -2.3)
Azerbaijan	605 (503 to 705)	11.86 (9.74 to 14)	1134 (866 to 1487)	12.66 (9.37 to 17.87)	0.23% (0.13 to 0.33)
Bahamas	62 (53 to 71)	36.96 (31.79 to 42.59)	115 (91 to 147)	29.43 (23.42 to 37.12)	-0.72% (-0.92 to -0.53)
Bahrain	91 (75 to 106)	73.56 (60.08 to 85.77)	182 (135 to 238)	30.65 (23.82 to 38.41)	-4.06% (-4.8 to -3.32)
Bangladesh	13971 (10584 to 17808)	28.47 (21.71 to 35.88)	32389 (21545 to 42778)	25.17 (16.95 to 33)	-0.16% (-0.36 to 0.04)
Barbados	44 (37 to 51)	15.37 (12.88 to 17.78)	60 (47 to 74)	12.68 (10.04 to 15.66)	-0.88% (-1.06 to -0.69)
Belarus	4845 (4070 to 6077)	37.99 (31.95 to 47.4)	8518 (6725 to 10604)	53.37 (42.09 to 66.72)	1.27% (1.08 to 1.45)
Belgium	7498 (6866 to 8087)	48.16 (44.36 to 51.67)	11030 (9659 to 12330)	45.83 (40.73 to 51.19)	-0.65% (-0.98 to -0.32)
Belize	10 (9 to 12)	9.75 (8.13 to 11.99)	32 (27 to 38)	11.05 (9.32 to 12.96)	0.22% (-0.05 to 0.48)
Benin	774 (378 to 1482)	18.05 (12.24 to 27.3)	1443 (939 to 2320)	18.77 (13.82 to 24.97)	0.06% (-0.08 to 0.19)
Bermuda	17 (14 to 21)	28.77 (24.02 to 35.14)	18 (14 to 23)	13.98 (11.11 to 17.68)	-2.83% (-3.04 to -2.63)
Bhutan	54 (33 to 83)	21.54 (14.66 to 32.14)	151 (95 to 292)	28.39 (18.2 to 54.22)	0.98% (0.86 to 1.1)
Bolivia (Plurinational State of)	1327 (693 to 2225)	31.63 (19.78 to 46.32)	2307 (1691 to 3003)	27.15 (20 to 34.94)	-0.7% (-0.83 to -0.56)
Bosnia and Herzegovina	709 (603 to 807)	19.57 (16.75 to 22.15)	1248 (955 to 1592)	21.08 (16.28 to 26.72)	0.16% (0.01 to 0.3)
Botswana	145 (96 to 225)	21.52 (14.39 to 32.7)	386 (267 to 532)	24.4 (17.31 to 33.32)	-0.19% (-0.63 to 0.25)
Brazil	55153 (52631 to 57576)	59.26 (56.15 to 62.01)	89267 (82618 to 96309)	38.06 (35.21 to 41.16)	-1.64% (-1.72 to -1.56)
Brunei Darussalam	30 (22 to 42)	28.25 (20.66 to 38.93)	73 (61 to 85)	27 (23.04 to 31.1)	-0.02% (-0.12 to 0.08)
Bulgaria	3124 (2801 to 3455)	26.1 (23.58 to 28.63)	4968 (3924 to 6172)	35.12 (27.63 to 43.87)	2.12% (1.63 to 2.62)
Burkina Faso	1705 (848 to 3327)	20.58 (14.77 to 29.53)	3473 (1789 to 6384)	24.2 (14.44 to 39.07)	0.83% (0.59 to 1.06)
Burundi	719 (413 to 1142)	26.58 (16.22 to 39.27)	1412 (934 to 2096)	29.58 (19.79 to 43.53)	0.2% (-0.04 to 0.43)
Cabo Verde	33 (25 to 43)	12.23 (9.29 to 15.33)	68 (55 to 81)	15.47 (12.62 to 18.54)	-0.03% (-0.44 to 0.37)
Cambodia	530 (369 to 785)	11.78 (8.8 to 15.44)	1335 (1047 to 1647)	12.64 (10 to 15.49)	0.19% (0.13 to 0.25)
Cameroon	1284 (798 to 2128)	18.11 (13.45 to 23.68)	3318 (1835 to 5367)	20.48 (12.15 to 32.22)	0.45% (0.3 to 0.6)
Canada	12059 (11199 to 12924)	37.62 (34.83 to 40.4)	21773 (18963 to 25000)	31.88 (28.1 to 36.28)	-0.57% (-0.65 to -0.5)
Central African Republic	548 (289 to 942)	42.78 (24.93 to 69.35)	933 (506 to 1612)	40.27 (24.45 to 64.7)	-0.17% (-0.27 to -0.08)
Chad	1219 (540 to 2322)	23.3 (14.15 to 35.53)	2326 (1210 to 4079)	24.25 (16.2 to 34.34)	0.11% (-0.01 to 0.23)
Chile	4983 (4569 to 5441)	51.32 (47.06 to 56.09)	8831 (7732 to 9877)	37.14 (32.48 to 41.48)	-1.16% (-1.24 to -1.08)

Measure	1990		2019		1990-2019
	Years (95% UI)	ASR (95% UI)	Years (95% UI)	ASR (95% UI)	EAPC (95% UI)
China	66074 (55137 to 85759)	8.32 (7.1 to 10.52)	118665 (96160 to 137360)	6.24 (5.09 to 7.18)	-0.93% (-0.98 to -0.87)
Colombia	7797 (7239 to 8335)	41.88 (38.72 to 44.68)	19056 (14535 to 24409)	36.35 (27.67 to 46.69)	-0.56% (-0.75 to -0.37)
Comoros	75 (41 to 115)	32.57 (19.4 to 47.38)	152 (105 to 212)	31.61 (21.94 to 43.67)	-0.27% (-0.4 to -0.14)
Congo	474 (297 to 712)	42.22 (28.26 to 59.84)	903 (618 to 1234)	35.44 (25.19 to 47.12)	-0.6% (-0.71 to -0.5)
Cook Islands	3 (2 to 4)	23.63 (18.97 to 30.73)	5 (4 to 6)	21.49 (16.87 to 27.33)	-0.38% (-0.48 to -0.29)
Costa Rica	982 (873 to 1099)	56.97 (50.23 to 63.8)	3081 (2344 to 3911)	61.09 (46.47 to 77.68)	-0.14% (-0.35 to 0.07)
Côte d'Ivoire	1693 (772 to 3250)	19.4 (11.71 to 29.05)	2799 (1757 to 4262)	18.66 (12.58 to 26.1)	-0.35% (-0.58 to -0.12)
Croatia	3389 (3071 to 3727)	54.49 (49.65 to 59.82)	3361 (2614 to 4178)	36.47 (28.39 to 45.56)	-1.72% (-2 to -1.43)
Cuba	4270 (3701 to 4930)	41.3 (35.83 to 47.59)	6962 (5490 to 8832)	37.24 (29.46 to 47.16)	-0.37% (-0.54 to -0.21)
Cyprus	198 (138 to 307)	28.06 (18.86 to 46.35)	349 (294 to 433)	19.17 (16.12 to 24.39)	-1.67% (-1.77 to -1.56)
Czechia	4838 (4462 to 5325)	35.16 (32.52 to 38.57)	4414 (3562 to 5505)	21 (17.01 to 26.02)	-1.71% (-1.83 to -1.58)
Democratic People's Republic of Korea	1253 (888 to 1707)	8.4 (6.16 to 11)	2443 (1644 to 3131)	7.85 (5.27 to 10)	-0.08% (-0.16 to -0.01)
Democratic Republic of the Congo	10195 (3649 to 19538)	55.92 (23.14 to 101.37)	15367 (7948 to 25927)	40.07 (20.66 to 67.02)	-1.31% (-1.53 to -1.09)
Denmark	2863 (2578 to 3169)	35.43 (31.87 to 39.23)	2503 (2176 to 2864)	21.23 (18.59 to 24.15)	-2.82% (-3.33 to -2.31)
Djibouti	43 (27 to 68)	26.58 (18.87 to 40.06)	160 (104 to 243)	27.18 (18.61 to 39.66)	-0.06% (-0.12 to -0.01)
Dominica	26 (23 to 30)	36.45 (31.48 to 42.17)	27 (22 to 33)	30.55 (24.5 to 38.07)	-0.54% (-0.61 to -0.48)
Dominican Republic	662 (566 to 811)	15.32 (13.22 to 18.58)	1495 (1051 to 2000)	15.9 (11.29 to 21.25)	0.53% (0.17 to 0.89)
Ecuador	1653 (1412 to 1917)	26.71 (23.4 to 30.64)	4438 (3492 to 5814)	30.29 (23.92 to 39.34)	1% (0.61 to 1.4)
Egypt	2986 (2378 to 4084)	11.12 (8.84 to 16.33)	6651 (3458 to 9940)	11.42 (5.97 to 17.98)	0.33% (0.21 to 0.46)
El Salvador	500 (441 to 558)	16.07 (14.25 to 17.88)	1082 (809 to 1406)	17.92 (13.35 to 23.38)	0.62% (0.44 to 0.81)
Equatorial Guinea	81 (39 to 153)	37.87 (18.94 to 67.39)	203 (112 to 337)	41.13 (23.02 to 67.19)	0.37% (0.26 to 0.48)
Eritrea	279 (157 to 448)	24.2 (16.79 to 34.04)	902 (634 to 1235)	33.39 (24.1 to 44.74)	0.93% (0.71 to 1.15)
Estonia	1635 (1466 to 1849)	79.39 (71.4 to 89.86)	1703 (1369 to 2134)	59.84 (47.67 to 75.47)	-1.45% (-1.89 to -1.02)
Eswatini	51 (37 to 77)	14.01 (10.22 to 21.07)	141 (95 to 205)	21.01 (14.5 to 29.89)	1.68% (1.44 to 1.93)
Ethiopia	8503 (4305 to 15011)	36.64 (23.22 to 58.47)	13717 (9573 to 18920)	31.81 (22.1 to 44.91)	-0.67% (-0.9 to -0.43)
Fiji	56 (44 to 72)	13.67 (10.82 to 17.31)	107 (80 to 140)	14.4 (10.96 to 18.51)	0.15% (0.07 to 0.23)
Finland	2646 (2295 to 3058)	36.14 (31.5 to 41.53)	3250 (2836 to 3635)	24.36 (21.5 to 27.07)	-1.63% (-1.78 to -1.48)
France	37410 (34286 to 40207)	43.36 (40.17 to 46.33)	42309 (36612 to 47877)	29.38 (25.69 to 33.3)	-1.44% (-1.5 to -1.38)
Gabon	224 (132 to 339)	38.43 (23.46 to 56.77)	362 (258 to 502)	35.44 (25.19 to 48.73)	-0.35% (-0.5 to -0.19)
Gambia	154 (87 to 273)	22.4 (15.27 to 32.03)	235 (165 to 315)	18.98 (13.8 to 25.22)	-1.04% (-1.3 to -0.77)
Georgia	508 (433 to 592)	8.81 (7.54 to 10.28)	1045 (807 to 1297)	17.47 (13.65 to 21.48)	3.51% (2.64 to 4.38)
Germany	66337 (59667 to 73421)	51.06 (45.9 to 56.3)	67665 (59599 to 76109)	34.35 (30.33 to 38.53)	-1.71% (-1.87 to -1.55)
Ghana	2653 (1702 to 4106)	23.53 (16.93 to 32.75)	4210 (2859 to 6052)	20.07 (14.89 to 26.65)	-1.06% (-1.52 to -0.59)
Greece	2474 (2250 to 2674)	16.58 (15.09 to 17.9)	4600 (3956 to 5305)	18.3 (16.02 to 20.75)	0.15% (-0.17 to 0.48)
Greenland	25 (20 to 32)	77.09 (61.66 to 96.41)	50 (39 to 61)	72.88 (57.35 to 90)	-0.46% (-0.66 to -0.26)
Grenada	9 (8 to 11)	12.63 (10.66 to 15.12)	15 (13 to 18)	13.79 (11.87 to 16.19)	0.32% (0.07 to 0.56)
Guam	9 (7 to 10)	12.93 (10.79 to 15.55)	22 (17 to 27)	11.85 (9.28 to 14.41)	-0.08% (-0.46 to 0.3)
Guatemala	954 (659 to 1296)	18.75 (15.35 to 22.54)	1616 (1268 to 2050)	13.82 (10.87 to 17.34)	-1.13% (-1.33 to -0.93)
Guinea	1296 (478 to 2661)	20.66 (11.1 to 34.93)	1588 (826 to 2681)	18.66 (11.38 to 28.4)	-0.37% (-0.46 to -0.28)

Measure	1990		2019		1990-2019
	Years (95% UI)	ASR (95% UI)	Years (95% UI)	ASR (95% UI)	EAPC (95% UI)
Guinea-Bissau	214 (79 to 441)	27.42 (14.48 to 46.24)	252 (162 to 385)	25.01 (17.44 to 35.59)	-0.28% (-0.39 to -0.17)
Guyana	100 (83 to 116)	23.76 (19.9 to 27.64)	150 (114 to 197)	23.04 (17.62 to 29.91)	0.19% (-0.04 to 0.42)
Haiti	1029 (636 to 1579)	29.67 (20.66 to 39.37)	1578 (1024 to 2375)	21.59 (14.48 to 30.64)	-0.97% (-1.15 to -0.78)
Honduras	1707 (1350 to 2134)	64.98 (51.52 to 78.91)	4510 (3218 to 5862)	78.19 (56.22 to 100.35)	0.91% (0.76 to 1.07)
Hungary	9656 (9117 to 10189)	67.88 (64.04 to 71.64)	9708 (7962 to 11849)	51.22 (41.71 to 62.87)	-1.34% (-1.53 to -1.16)
Iceland	88 (77 to 98)	30.33 (26.93 to 33.69)	101 (85 to 116)	17.85 (15.25 to 20.59)	-2.34% (-2.58 to -2.11)
India	88437 (66851 to 121507)	20.8 (16.19 to 27.41)	232036 (160044 to 331574)	21.4 (14.87 to 30.26)	-0.13% (-0.25 to -0.01)
Indonesia	15264 (12414 to 18804)	17.51 (14.35 to 21.03)	33090 (27287 to 39300)	19.35 (16.03 to 22.71)	0.27% (0.2 to 0.34)
Iran (Islamic Republic of)	2526 (2050 to 3161)	10.85 (7.99 to 14.5)	6274 (5188 to 7062)	9.03 (7.49 to 10.17)	-0.34% (-0.66 to -0.02)
Iraq	803 (577 to 1121)	8.81 (6.67 to 11.6)	2000 (1531 to 2513)	7.98 (6.09 to 9.77)	-0.19% (-0.34 to -0.03)
Ireland	1325 (1224 to 1441)	32.12 (29.77 to 34.8)	2144 (1852 to 2420)	28.53 (24.71 to 32.14)	-0.01% (-0.2 to 0.18)
Israel	1617 (1470 to 1769)	33.34 (30.34 to 36.38)	3353 (2895 to 3877)	28.09 (24.39 to 32.23)	-1% (-1.14 to -0.85)
Italy	27009 (25314 to 28134)	30.45 (28.6 to 31.72)	29999 (26194 to 32425)	19.15 (17.22 to 20.53)	-1.78% (-1.93 to -1.64)
Jamaica	260 (221 to 300)	14.06 (12.04 to 16.12)	353 (269 to 451)	11.86 (9.04 to 15.19)	-0.66% (-1.06 to -0.26)
Japan	21963 (20710 to 23230)	13.46 (12.65 to 14.24)	46930 (39375 to 52273)	12.76 (11.26 to 13.92)	-0.1% (-0.21 to 0.02)
Jordan	401 (312 to 505)	29.68 (23.19 to 37.47)	1072 (853 to 1331)	16.59 (13.3 to 20.38)	-2.58% (-2.94 to -2.23)
Kazakhstan	4187 (3714 to 4886)	34.24 (30.4 to 40.31)	5092 (4293 to 6141)	30.07 (25.46 to 36)	-0.84% (-1.25 to -0.43)
Kenya	2728 (1671 to 4218)	32.09 (18.27 to 50.93)	8410 (4961 to 12703)	40.69 (23.2 to 62.39)	0.99% (0.92 to 1.06)
Kiribati	24 (16 to 35)	55.39 (40.12 to 73.17)	40 (27 to 57)	49.48 (35.53 to 69.3)	-0.38% (-0.43 to -0.33)
Kuwait	145 (127 to 165)	21.4 (18.25 to 24.86)	406 (331 to 491)	17.22 (13.8 to 20.71)	-0.38% (-0.67 to -0.1)
Kyrgyzstan	442 (394 to 494)	13.74 (12.24 to 15.3)	479 (407 to 564)	10.4 (8.85 to 12.09)	-1.39% (-1.62 to -1.16)
Lao People's Democratic Republic	225 (139 to 345)	10.89 (7.37 to 15.49)	463 (329 to 623)	11.41 (8.19 to 15.08)	0.02% (-0.09 to 0.12)
Latvia	2922 (2650 to 3254)	80.95 (73.54 to 89.89)	2974 (2469 to 3560)	69.43 (57.72 to 83.57)	-0.99% (-1.33 to -0.66)
Lebanon	255 (180 to 326)	12.24 (8.58 to 15.69)	580 (357 to 822)	11.08 (6.78 to 15.71)	-0.24% (-0.36 to -0.13)
Lesotho	149 (104 to 214)	13.96 (9.8 to 19.98)	268 (183 to 385)	19.56 (13.76 to 27.17)	1.47% (1.28 to 1.66)
Liberia	457 (198 to 880)	23.2 (13.27 to 37.58)	494 (295 to 879)	17.99 (12.16 to 26.64)	-0.5% (-0.83 to -0.16)
Libya	241 (147 to 361)	13.52 (7.74 to 20.86)	541 (364 to 774)	11.22 (7.51 to 16.09)	-0.58% (-0.65 to -0.51)
Lithuania	2090 (1883 to 2323)	46.36 (41.78 to 51.48)	4418 (3539 to 5433)	71.34 (57.18 to 87.8)	1.91% (1.58 to 2.25)
Luxembourg	293 (263 to 323)	53.66 (48.53 to 58.86)	366 (302 to 434)	35.4 (29.53 to 42.03)	-1.8% (-2.04 to -1.56)
Madagascar	1222 (695 to 1720)	20.23 (12.81 to 29.39)	2691 (1952 to 3671)	24.52 (17.77 to 33.83)	0.71% (0.6 to 0.83)
Malawi	1192 (830 to 1778)	25.77 (18.88 to 32.82)	2211 (1557 to 2992)	29.1 (19.98 to 39.94)	0.42% (0.31 to 0.53)
Malaysia	1541 (1299 to 1839)	16.23 (13.7 to 19.34)	6505 (4929 to 8494)	26.6 (20.06 to 35.08)	1.44% (1.15 to 1.73)
Maldives	11 (5 to 21)	12.04 (6.76 to 19.98)	26 (21 to 31)	9.42 (7.63 to 11.6)	-1.34% (-1.49 to -1.18)
Mali	1722 (560 to 3767)	20.09 (10.45 to 33.56)	3033 (1426 to 5649)	20.79 (12.8 to 31.94)	0.21% (0.09 to 0.32)
Malta	111 (99 to 124)	26.47 (23.59 to 29.4)	191 (159 to 222)	20.19 (17.05 to 23.45)	-1.31% (-1.6 to -1.02)
Marshall Islands	3 (2 to 5)	18.83 (13.28 to 25.07)	7 (4 to 11)	18.91 (11.41 to 30.17)	-0.05% (-0.2 to 0.11)
Mauritania	316 (228 to 477)	21.77 (16.29 to 28.63)	375 (243 to 581)	15.74 (10.82 to 23.01)	-1.33% (-1.46 to -1.19)
Mauritius	55 (49 to 61)	7.71 (6.87 to 8.63)	145 (113 to 178)	8.88 (6.97 to 10.87)	0.48% (0.28 to 0.69)
Mexico	21464 (20496 to 22274)	47.77 (45.4 to 49.59)	47173 (40470 to 54757)	41.44 (35.43 to 48.01)	-0.61% (-0.73 to -0.5)

Measure	1990		2019		1990-2019
	Years (95% UI)	ASR (95% UI)	Years (95% UI)	ASR (95% UI)	EAPC (95% UI)
Micronesia (Federated States of)	9 (6 to 12)	18.22 (13.78 to 23.9)	14 (8 to 25)	20.08 (11.76 to 34.2)	0.35% (0.24 to 0.47)
Monaco	18 (14 to 22)	24.83 (19.43 to 30.63)	24 (19 to 29)	24.47 (19.25 to 29.68)	-0.02% (-0.09 to 0.05)
Mongolia	153 (124 to 187)	14.14 (11.4 to 17.29)	258 (198 to 333)	11.94 (9.24 to 15.25)	-0.84% (-0.96 to -0.72)
Montenegro	504 (396 to 628)	81.45 (64.25 to 101.48)	843 (682 to 1041)	87.99 (71.4 to 108.88)	0.38% (0.23 to 0.52)
Morocco	1579 (1077 to 2111)	12.52 (8.28 to 17.61)	3492 (2622 to 4427)	12.36 (9.33 to 15.39)	-0.04% (-0.18 to 0.09)
Mozambique	1954 (1277 to 2764)	29.02 (21.66 to 36.71)	5271 (3665 to 7389)	44.33 (31.38 to 61.16)	1.78% (1.63 to 1.93)
Myanmar	1709 (902 to 3166)	6.66 (3.76 to 11.37)	2911 (2034 to 4360)	6.39 (4.55 to 9.29)	-0.14% (-0.27 to 0)
Namibia	169 (118 to 223)	21.27 (15.09 to 27.85)	364 (256 to 512)	23.57 (17.2 to 32.13)	0.32% (0.15 to 0.49)
Nauru	1 (1 to 2)	20.5 (12.97 to 30.57)	1 (1 to 2)	21.6 (13.81 to 32.93)	0.14% (-0.04 to 0.33)
Nepal	2910 (2127 to 4017)	32.26 (22.9 to 45.12)	6470 (4757 to 9135)	31.93 (23.76 to 44.56)	-0.31% (-0.44 to -0.18)
Netherlands	7065 (6542 to 7551)	35.28 (32.71 to 37.69)	9609 (8514 to 10891)	28.13 (25.1 to 31.59)	-1.11% (-1.39 to -0.83)
New Zealand	1301 (1195 to 1422)	32.84 (30.26 to 35.79)	1609 (1393 to 1860)	20.14 (17.62 to 23.16)	-1.82% (-2.14 to -1.51)
Nicaragua	229 (200 to 255)	14.26 (12.37 to 15.95)	757 (610 to 954)	17.96 (14.58 to 22.23)	0.76% (0.51 to 1.02)
Niger	1887 (506 to 4537)	24.43 (12.26 to 42.89)	3104 (1415 to 6097)	22.12 (13.35 to 34.23)	-0.42% (-0.58 to -0.27)
Nigeria	16335 (11137 to 24114)	22.33 (15.54 to 32.74)	28540 (16643 to 47634)	22.85 (13.13 to 39.96)	0.3% (0.14 to 0.45)
Niue	0 (0 to 0)	15.34 (11.69 to 19.83)	0 (0 to 0)	17.31 (12.01 to 23)	0.44% (0.32 to 0.57)
North Macedonia	698 (542 to 802)	37.54 (29.33 to 43.35)	805 (616 to 1027)	25.79 (20.02 to 32.5)	-1.82% (-2.1 to -1.54)
Northern Mariana Islands	5 (4 to 6)	24.24 (19.91 to 29.92)	10 (8 to 12)	19.85 (15.87 to 23.77)	-0.66% (-0.83 to -0.48)
Norway	1823 (1686 to 2014)	25.52 (23.77 to 28)	1723 (1543 to 1965)	17.28 (15.63 to 19.5)	-1.78% (-1.96 to -1.6)
Oman	75 (53 to 105)	12.67 (8.93 to 17.68)	165 (126 to 221)	11.46 (9.41 to 13.72)	0.03% (-0.1 to 0.17)
Pakistan	14546 (9256 to 21693)	25.91 (16.27 to 40.04)	27854 (17974 to 39502)	27.35 (17.96 to 37.34)	0.26% (0.18 to 0.35)
Palau	1 (1 to 2)	13.41 (9.02 to 20.22)	3 (2 to 5)	16.03 (9.39 to 22.71)	0.71% (0.58 to 0.83)
Palestine	135 (89 to 203)	15.3 (10.85 to 21.09)	329 (267 to 401)	15.42 (12.52 to 18.71)	-0.08% (-0.21 to 0.06)
Panama	447 (396 to 500)	28.08 (24.73 to 31.41)	1022 (775 to 1317)	24.55 (18.6 to 31.68)	-0.57% (-0.75 to -0.39)
Papua New Guinea	144 (100 to 203)	6.37 (4.57 to 8.28)	479 (328 to 697)	8.16 (5.89 to 11.11)	0.94% (0.82 to 1.06)
Paraguay	664 (550 to 777)	29.11 (23.71 to 34.11)	1850 (1354 to 2516)	33.83 (24.8 to 46.18)	0.83% (0.68 to 0.98)
Peru	4032 (2680 to 6047)	24 (17.96 to 31.85)	3732 (2729 to 5021)	11.57 (8.45 to 15.57)	-2.84% (-3.19 to -2.49)
Philippines	2179 (1773 to 2643)	7.47 (5.86 to 9.17)	5068 (4190 to 5988)	6.96 (5.78 to 8.22)	-0.31% (-0.55 to -0.06)
Poland	8334 (7902 to 8761)	19.34 (18.32 to 20.37)	23918 (20208 to 27911)	34.2 (28.82 to 39.97)	1.6% (0.61 to 2.61)
Portugal	4602 (4210 to 4970)	34.32 (31.47 to 37.03)	11165 (9739 to 12488)	43.31 (38.24 to 48.03)	0.8% (0.52 to 1.07)
Puerto Rico	786 (708 to 868)	21.81 (19.66 to 24.08)	828 (622 to 1055)	12.26 (9.23 to 15.73)	-2.79% (-3.06 to -2.51)
Qatar	17 (13 to 22)	17.16 (13.1 to 22.86)	110 (77 to 152)	15.66 (11.43 to 21.17)	-0.02% (-0.22 to 0.18)
Republic of Korea	3316 (2549 to 4666)	11.49 (8.95 to 15.78)	10298 (7891 to 12097)	12.22 (9.43 to 14.41)	0.84% (0.57 to 1.1)
Republic of Moldova	1813 (1648 to 1981)	40.68 (37.09 to 44.31)	3773 (3167 to 4433)	66.04 (55.42 to 77.35)	2.39% (1.98 to 2.8)
Romania	11084 (10125 to 12125)	40.9 (37.48 to 44.52)	18527 (15216 to 22631)	52.08 (42.62 to 63.54)	1.03% (0.86 to 1.21)
Russian Federation	107401 (91046 to 128888)	60.82 (51.57 to 72.62)	193248 (169710 to 218877)	83.17 (72.96 to 94.12)	1.16% (0.91 to 1.41)
Rwanda	1067 (703 to 1434)	33.55 (20.76 to 48.31)	1702 (899 to 2610)	28.45 (15.27 to 42.76)	-1.13% (-1.45 to -0.82)
Saint Kitts and Nevis	17 (14 to 21)	45.9 (38 to 57.06)	20 (16 to 26)	31.99 (25.22 to 41.02)	-1.36% (-1.54 to -1.18)
Saint Lucia	24 (21 to 28)	27.19 (23.4 to 31.11)	45 (36 to 55)	21.39 (17.38 to 26.09)	-1.2% (-1.55 to -0.85)

Measure	1990		2019		1990-2019
	Years (95% UI)	ASR (95% UI)	Years (95% UI)	ASR (95% UI)	EAPC (95% UI)
Saint Vincent and the Grenadines	8 (7 to 9)	10.36 (9.11 to 11.72)	11 (9 to 13)	8.08 (6.65 to 9.62)	-0.73% (-1.12 to -0.35)
Samoa	12 (9 to 15)	13.37 (10.41 to 17.44)	21 (16 to 27)	14.2 (10.89 to 17.96)	0.32% (0.26 to 0.39)
San Marino	3 (2 to 3)	8.24 (6.75 to 9.79)	6 (4 to 8)	8.32 (5.52 to 11.98)	0.32% (0.2 to 0.43)
Sao Tome and Principe	21 (13 to 35)	19.39 (13.13 to 27.24)	18 (12 to 31)	14.7 (10.38 to 24.54)	-1.04% (-1.31 to -0.78)
Saudi Arabia	968 (646 to 1416)	17.6 (11.83 to 25.7)	2128 (1431 to 3198)	12.65 (8.87 to 21.47)	-1.36% (-1.47 to -1.24)
Senegal	1540 (586 to 3168)	24.5 (12.87 to 40.06)	1974 (1152 to 2998)	21.79 (13.24 to 33.25)	-0.49% (-0.67 to -0.31)
Serbia	6341 (5230 to 7812)	58.25 (48.04 to 70.3)	7880 (6242 to 9811)	48.68 (38.75 to 60.3)	-0.73% (-1.02 to -0.45)
Seychelles	6 (5 to 8)	11.26 (8.14 to 14.43)	13 (10 to 18)	13.13 (9.37 to 18.07)	0.11% (-0.15 to 0.37)
Sierra Leone	674 (271 to 1387)	18.56 (10.6 to 31.28)	1016 (588 to 1716)	18.99 (12.34 to 28.61)	0.27% (-0.02 to 0.56)
Singapore	304 (273 to 340)	13.7 (12.33 to 15.25)	1101 (947 to 1243)	14.99 (12.88 to 16.9)	1.05% (0.61 to 1.49)
Slovakia	871 (760 to 1001)	14.69 (12.85 to 16.86)	2094 (1612 to 2707)	23.01 (17.74 to 29.74)	2.3% (1.98 to 2.62)
Slovenia	1446 (1131 to 1844)	59.5 (46.48 to 75.68)	1837 (1416 to 2415)	39.23 (30.19 to 51.59)	-1.65% (-1.91 to -1.4)
Solomon Islands	16 (10 to 24)	10.1 (6.93 to 14.05)	41 (27 to 61)	11.6 (8.23 to 15.88)	0.5% (0.35 to 0.66)
Somalia	637 (284 to 1109)	21.92 (11.77 to 32.77)	1479 (854 to 2367)	20.73 (12.58 to 30.35)	-0.16% (-0.22 to -0.11)
South Africa	4000 (3215 to 4818)	16 (12.78 to 19.99)	7887 (6642 to 9161)	16.9 (14.23 to 19.58)	0.13% (-0.08 to 0.34)
South Sudan	827 (436 to 1375)	28.92 (18.41 to 47.27)	1177 (755 to 2051)	28.77 (18.75 to 49.37)	-0.01% (-0.05 to 0.02)
Spain	26768 (24856 to 28501)	48.99 (45.48 to 52.11)	43744 (37840 to 50493)	40.48 (35.66 to 45.62)	-0.98% (-1.34 to -0.62)
Sri Lanka	888 (681 to 1150)	8.13 (6.27 to 10.62)	1805 (1328 to 2387)	7.59 (5.61 to 9.94)	-0.09% (-0.29 to 0.11)
Sudan	960 (494 to 1388)	10.19 (5.07 to 15.58)	1983 (1195 to 3166)	10.96 (6.13 to 18.14)	0.31% (0.26 to 0.37)
Suriname	32 (28 to 36)	11.98 (10.48 to 13.36)	66 (53 to 81)	11.36 (9.17 to 13.8)	-0.34% (-0.51 to -0.17)
Sweden	4879 (4452 to 5257)	30.36 (27.93 to 32.63)	3589 (3152 to 4003)	15.95 (14.12 to 17.73)	-2.4% (-2.48 to -2.31)
Switzerland	1841 (1607 to 2162)	17.1 (14.98 to 19.87)	3327 (2817 to 4004)	17.85 (15.31 to 21.04)	-0.01% (-0.32 to 0.31)
Syrian Arab Republic	449 (316 to 584)	9.04 (6.21 to 11.95)	872 (602 to 1173)	7.9 (5.54 to 10.35)	-0.62% (-0.76 to -0.48)
Taiwan (Province of China)	1251 (1148 to 1371)	8.45 (7.73 to 9.28)	8959 (7042 to 11419)	23.34 (18.44 to 29.72)	3.92% (2.98 to 4.87)
Tajikistan	429 (323 to 614)	11.69 (8.86 to 14.08)	758 (560 to 1040)	16 (12.13 to 21.73)	1.38% (1.27 to 1.48)
Thailand	4493 (3759 to 5212)	13.16 (10.99 to 15.51)	11682 (8772 to 15765)	12.12 (9.14 to 16.29)	-0.52% (-0.67 to -0.38)
Timor-Leste	24 (16 to 33)	8.33 (5.68 to 11.78)	80 (52 to 121)	10.44 (6.91 to 15.6)	0.92% (0.81 to 1.04)
Togo	522 (269 to 950)	19.55 (13.45 to 27.52)	981 (648 to 1409)	21.55 (14.67 to 30.09)	0.34% (0.13 to 0.55)
Tokelau	0 (0 to 0)	12.91 (9.14 to 18.23)	0 (0 to 0)	14.48 (10.89 to 18.94)	0.55% (0.45 to 0.65)
Tonga	10 (8 to 14)	18.62 (13.61 to 24.69)	17 (13 to 22)	20.6 (15.79 to 27.05)	0.33% (0.19 to 0.48)
Trinidad and Tobago	224 (197 to 255)	26.18 (22.94 to 29.78)	284 (207 to 377)	15.95 (11.66 to 21.06)	-2.42% (-2.76 to -2.08)
Tunisia	474 (329 to 655)	10 (6.89 to 13.68)	1195 (794 to 1692)	9.98 (6.7 to 13.96)	0.06% (0.03 to 0.09)
Turkey	7790 (5874 to 10152)	21.55 (16.46 to 27.17)	12913 (10281 to 15759)	15.01 (12.02 to 18.35)	-1.23% (-1.38 to -1.08)
Turkmenistan	287 (254 to 322)	13.61 (12.26 to 15.01)	709 (554 to 903)	18.28 (14.39 to 22.88)	0.97% (0.76 to 1.19)
Tuvalu	1 (1 to 2)	15.38 (10.43 to 21.88)	2 (1 to 2)	15.91 (11.14 to 22.4)	0.08% (-0.01 to 0.17)
Uganda	1984 (1332 to 2821)	27.53 (17.91 to 37.53)	4972 (3406 to 6857)	32.71 (22.85 to 43.75)	0.43% (0.3 to 0.55)
Ukraine	39122 (35560 to 42998)	55.27 (50.54 to 60.67)	48824 (40875 to 57439)	64.64 (54.18 to 76.24)	0.22% (0.04 to 0.41)
United Arab Emirates	85 (41 to 141)	18.79 (8.33 to 31.68)	695 (397 to 1172)	15.21 (8.91 to 28.09)	-0.91% (-1.35 to -0.46)
United Kingdom	31331 (27857 to 32844)	35.05 (31.58 to 36.7)	55098 (50591 to 60176)	43.77 (40.59 to 47.47)	0.91% (0.8 to 1.02)

Measure	1990		2019		1990-2019
	Years (95% UI)	ASR (95% UI)	Years (95% UI)	ASR (95% UI)	EAPC (95% UI)
United Republic of Tanzania	3013 (2243 to 4201)	23.57 (17.98 to 29.34)	7880 (5524 to 10537)	29.3 (20.69 to 39.54)	0.89% (0.76 to 1.03)
United States of America	138292 (129303 to 144016)	43.36 (40.73 to 45.1)	190067 (176529 to 199919)	35.11 (32.8 to 36.78)	-1.05% (-1.23 to -0.87)
United States Virgin Islands	20 (17 to 25)	24.16 (19.78 to 29.09)	36 (29 to 42)	20.36 (16.37 to 24.24)	-0.53% (-0.75 to -0.31)
Uruguay	3345 (3018 to 3658)	85.76 (77.67 to 93.62)	4381 (3857 to 4907)	78.11 (69.45 to 87.15)	-0.38% (-0.46 to -0.29)
Uzbekistan	1152 (888 to 1362)	8.97 (6.4 to 11.15)	2321 (1932 to 2757)	12.59 (10.6 to 14.91)	1.27% (1.18 to 1.36)
Vanuatu	8 (5 to 11)	11.66 (7.99 to 17.32)	29 (20 to 40)	15.91 (11.35 to 21.97)	1.16% (1.01 to 1.32)
Venezuela (Bolivarian Republic of)	4556 (4074 to 5166)	42.94 (38.2 to 48.38)	13003 (9610 to 16809)	46.12 (34.08 to 59.48)	0.33% (0.17 to 0.49)
Viet Nam	6407 (4797 to 8528)	17.07 (12.74 to 23.05)	10195 (7673 to 13158)	12.53 (9.39 to 16.41)	-1.48% (-1.68 to -1.27)
Yemen	640 (400 to 944)	13.92 (8.57 to 22.49)	1662 (1177 to 2385)	12.98 (8.97 to 19.17)	-0.16% (-0.2 to -0.11)
Zambia	1122 (707 to 1693)	32.12 (25.18 to 40.37)	2243 (1681 to 2946)	30.84 (23.15 to 40.18)	-0.35% (-0.5 to -0.19)
Zimbabwe	413 (303 to 569)	10.72 (7.62 to 14.94)	928 (485 to 1518)	14.14 (7.55 to 23.52)	1.02% (0.96 to 1.09)

UI=uncertainty interval; DALYs= disability-adjusted life years; ASR= age-standardized rates; EAPC= estimated annual percentage changes.

Vascular intestinal disorders

Flowchart

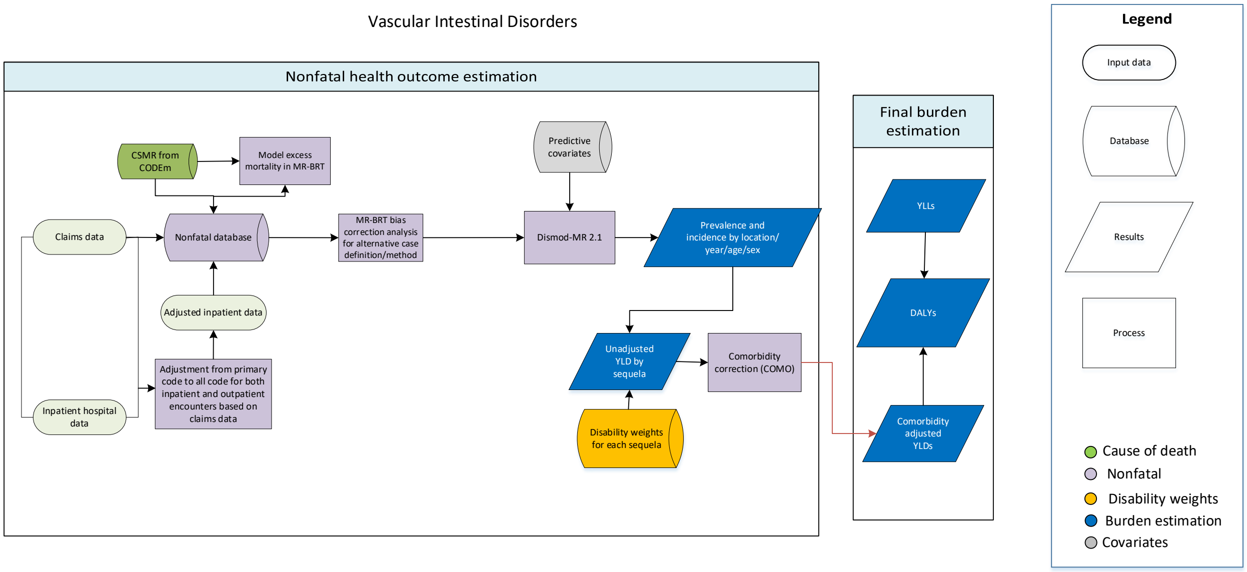

Input Data and Methodological Summary for Vascular Intestinal Disorders

Case definition

Vascular intestinal disorders comprise ischaemic disorders and vascular malformations (ie, angiodysplasias). Ischaemia occurs when there is decreased blood supply to the gastrointestinal tract, causing injury to the bowel, and vascular malformations occur when blood vessels in the bowel grow inappropriately, predisposing to bleeding. Vascular intestinal disorders typically require surgical treatment. The ICD10 code for vascular intestinal disorders is K55; ischaemia and angiodysplasia are only distinguished at the level of 4-digit and 5-digit codes. Equivalent codes for ICD9 are 569.84, 569.85 and 569.86 (for angiodysplasia), and 557 and its 4- and 5-digit constituents (for ischaemia).

Input data and data processing

Input data

Like GBD 2017, the model included data from hospital discharges and claims. In GBD 2019, we newly added Poland claims data and additional years of data from USA claims (years 2015-2016) and hospital discharges in Mexico, India, New Zealand, Sweden, Georgia, and Ecuador. Notably, we included hospital data from Botswana; southern sub-Saharan Africa previously did not have data.

Table 1. Data Inputs for vascular intestinal disorders morbidity modelling by parameter.

Measure	Total sources	Countries with data
Incidence	294	43

Data processing

Hospital discharge data provide observations about encounters, generally with only the primary diagnostic code for the encounter. Claims data, on the other hand, link claims for all inpatient and outpatient encounters for a single individual, and provide primary and secondary diagnoses for all encounters.

In GBD 2017, an individual was extracted from claims data as an incident case if that individual had one or more inpatient encounters with an appropriate ICD code as any diagnosis; readmissions within 28 days were assumed to be for the same episodes of illness. Data from hospital discharges with appropriate ICD codes as primary diagnostic code were, then, adjusted using correction factors derived from inpatient claims data, estimating the number of individuals represented by each encounter, and adjusting the number of individuals with vascular intestinal disorders as primary diagnostic code to the number expected if information on all diagnoses had been provided.

In GBD 2019, however, we improved data processing methods to capture cases that were diagnosed and/or treated in an outpatient setting. Specifically, incident cases were extracted from claim data if an individual had at least one inpatient or outpatient encounter with an appropriate ICD code as any diagnosis within one year. Data from hospital discharges were adjusted using correction factors from claims, converting encounters to estimates of cases, correcting for most locations providing only primary diagnostic codes, and estimating outpatient cases from inpatient cases.

The USA claims data from the year 2000 and from the years 2010–2016 were each adjusted to data from hospital discharges outside DisMod using MR-BRT analysis to adjust for selection bias due to commercial insurance.

The process of adjusting for non-reference data using MR-BRT with the logit-transformation method is described below:

1. Identify data points with overlapping year, age, sex, and location between claims (alternative case definition) and hospital discharges (reference case definition)
2. Logit transform overlapping data points of alternative and reference case definitions
3. Convert overlapping data points into a difference in logit space using the following equation: $logit(altnerative) - logit(reference)$
4. Use the delta method to compute standard errors of overlapping data points in logit space, then calculate standard error of logit difference using the following equation: $\sqrt{(variance\ of\ alternative) + (variance\ of\ reference)}$
5. Using MR-BRT, conduct a random effects meta-regression to obtain the pooled logit difference of alternative to reference
6. Apply the pooled logit difference to all data points of alternative case definitions using the following equation:
$$new_{estimate} = inverse.logit((logit(alternative)) - (pooled\ logit\ difference))$$
7. Calculate new standard errors using the delta method, accounting for gamma (between-study heterogeneity)

The table below shows bias correction factors estimated using MR-BRT.

Table 2. MR-BRT Crosswalk Adjustment Factors for Vascular Intestinal Disorders

Data input	Reference or alternative data collection	Gamma	Beta Coefficient, Logit (95% CI)	Adjustment factor*
Hospital + non-USA claims	Ref	0.05	---	---
USA claims from year 2000	Alt		-0.24 (-0.71, 0.22)	0.44 (0.33, 0.55)
USA claims from year 2010-2016	Alt		0.12 (-0.02, 0.26)	0.53 (0.50, 0.56)

**Adjustment factor is the inverse-logit transformed Beta coefficient; <0.5 represents that alternative is adjusted upward; >0.5 represents that alternative is adjusted downward*

Data points with an age-standardised incidence rate greater than three median absolute deviations from the median of the age-standardised incidence rate for all inpatient and non-USA claims data were marked as outliers and excluded from analysis.

Severity split & disability weight

The basis of the GBD disability weight survey assessments are lay descriptions of sequelae highlighting major functional consequences and symptoms. The lay description and disability weights for vascular intestinal disorders are shown below. All cases are assumed to be severe.

Table 3. Severity Distribution, details on the severity levels for vascular intestinal disorders in GBD 2019 and the associated disability weight (DW) with that severity.

Severity level	Lay description	DW (95% CI)
Severe	This person has severe pain in the belly and feels nauseated. The person is anxious and unable to carry out daily activities.	0.324 (0.219–0.442)

Modeling strategy

Similar to GBD 2017, we ran a DisMod-MR 2.1 model to produce estimates by age, sex, year, and country. Prior settings of the DisMod model included bounding remission between 2 and 12 (a duration from about four weeks to half a year) for all age groups. We used the function in DisMod to pull in cause-specific mortality rate (CSMR) data from our CODEm and CODcorrect analyses. The minimum coefficient of variation at the regional, super-regional, and global-level was changed from 0.4 to 0.8 in GBD 2019 to improve model fit against input data.

In previous rounds, priors on excess mortality rate (EMR) were estimated in DisMod by matching prevalence data points with their corresponding CSMR values within the same age, sex, year, and location (by dividing CSMR by prevalence). For short duration conditions (remission >1), the corresponding prevalence was derived by running an initial model and then applying the same CSMR/prevalence method. However, for many causes, DisMod estimated a rather unrealistic pattern of EMR compared to an expected pattern of decreasing EMR with greater access to quality health care. Such unexpected patterns often signal inconsistencies between CSMR estimates and the measures of

prevalence and/or incidence. In effort to provide greater guidance to DisMod on the expected pattern of EMR, EMR data generated in the previous round were modeled using the MR-BRT approach by age and sex with a prior on healthcare access and quality index (HAQi) having a negative coefficient. Results from MR-BRT were then predicted for each location, year, sex, and for ages 0, 10, 20….100. We included HAQi as a country-level covariate to inform EMR with a mean and standard deviation produced from MR-BRT.

A lag-distributed income covariate (log transformed) and a mean total cholesterol covariates were applied to incidence as predictive covariates. Betas and exponentiated values (which can be interpreted as an odds ratio) are shown in the table below.

Table 4. Covariates. Summary of covariates used in the vascular intestinal disorders DisMod-MR meta-regression model

Covariate	Type	Parameter	Exponentiated beta (95% Uncertainty Interval)
Cholesterol (total, mean per capita)	Country-level	Incidence	1.24 (1.17, 1.33)
LDI (I$ per capita)	Country-level	Incidence	1.21 (1.18, 1.22)
Healthcare access and quality index	Country-level	Excess mortality rate	0.96 (0.96, 0.96)
